# Assessing the Interactions between Snake Venom Metalloproteinases and Hydroxamate Inhibitors Using Kinetic and ITC Assays, Molecular Dynamics Simulations and MM/PBSA-Based Scoring Functions

**DOI:** 10.1021/acsomega.4c08439

**Published:** 2024-12-10

**Authors:** Raoni A. de Souza, Natalia Díaz, Luis G. Fuentes, Adriano Pimenta, Ronaldo A. P. Nagem, Carlos Chávez-Olórtegui, Francisco S. Schneider, Franck Molina, Eladio F. Sanchez, Dimas Suárez, Rafaela S. Ferreira

**Affiliations:** † Rua Conde Pereira Carneiro 80, Dept. de Pesquisa e Desenvolvimento, Fundação Ezequiel Dias, Belo Horizonte 30510-010, Minas Gerais, Brazil; ‡ Avda Julián Clavería 8, Dept. de Química Física y Analítica, Universidad de Oviedo, Oviedo 33006, Asturias, Spain; § Carretera Sacramento s/n, Dept. de Química y Física, Universidad de Almería, Almería 04120, Andalucía, Spain; ∥ Avenida Antônio Carlos 6627, Dept. De Bioquímica e Imunologia, Universidade Federal de Minas Gerais, Belo Horizonte 31270-901, Minas Gerais, Brazil; ⊥ 1682, Rue de la Valsière, Sys2Diag (UMR9005 CNRS − ALCEN), Cap Delta, Montpellier 34184, Occitanie, France

## Abstract

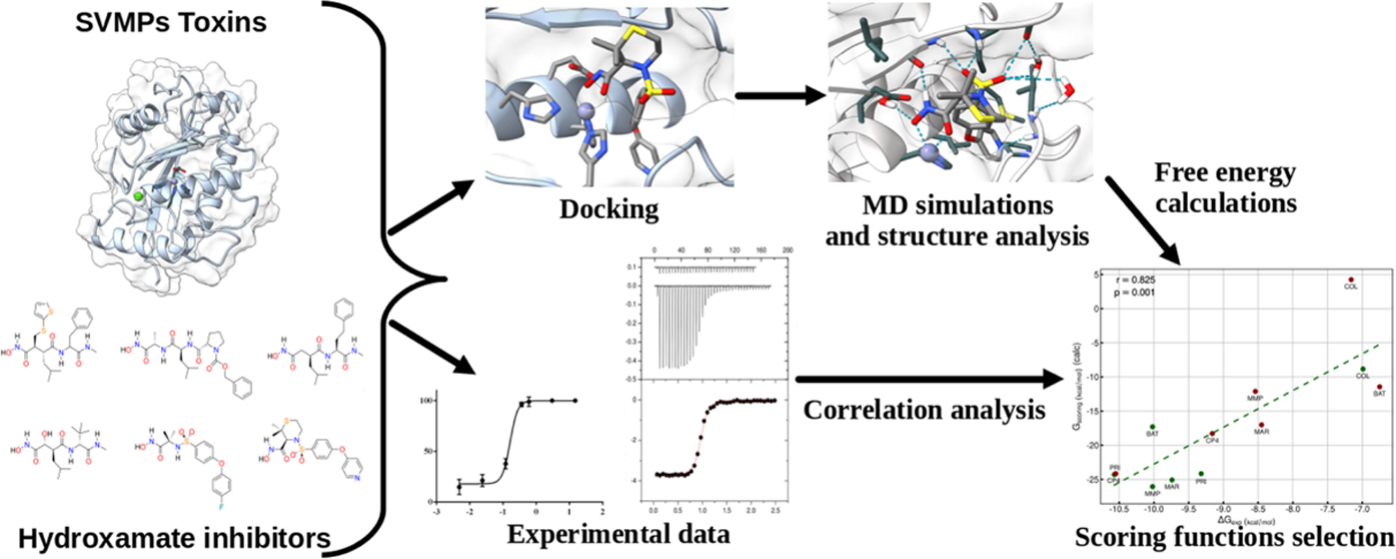

*Bothrops* species are the main cause of snake bites in rural communities of tropical developing countries of Central and South America. Envenomation by *Bothrops* snakes is characterized by prominent local inflammation, hemorrhage and necrosis as well as systemic hemostatic disturbances. These pathological effects are mainly caused by the major toxins of the viperidae venoms, the snake venom metalloproteinases (SVMPs). Despite the antivenom therapy efficiency to block the main toxic effects on bite victims, this treatment shows limited efficacy to prevent tissue necrosis. Thus, drug-like inhibitors of these toxins have the potential to aid serum therapy of accidents inflicted by viper snakes. Broad-spectrum metalloprotease inhibitors bearing a hydroxamate zinc-binding group are potential candidates to improve snake bites therapy and could also be used to study toxin-ligand interactions. Therefore, in this work, we used both docking calculations and molecular dynamics simulations to assess the interactions between six hydroxamate inhibitors and two P–I SVMPs selected as models: Atroxlysin-I (hemorrhagic) from *Bothrops atrox*, and Leucurolysin-a (nonhemorrhagic) from *Bothrops leucurus*. We also employed a large variety of end-point free energy methods in combination with entropic terms to produce scoring functions of the relative affinities of the inhibitors for the toxins. Then we identified the scoring functions that best correlated with experimental data obtained from kinetic activity assays. In addition, to the characterization of these six molecules as inhibitors of the toxins, this study sheds light on the main enzyme–inhibitor interactions, explaining the broad-spectrum behavior of the inhibitors, and identifies the energetic and entropic terms that improve the performance of the scoring functions.

## Introduction

Snake bites are neglected tropical diseases (NTD) that represent an important public health problem,^1^ causing approximately 125,000 deaths worldwide every year.^2^ Current estimates are that each year snake bites cause up to 130–150 thousand cases in Latin America^3^ and approximately 30,000 in Brazil,^4^ that are caused mainly by viperidae venomous snakes (subfamily crotalinae, “pit vipers”).^3,5,6^ These numbers are likely to be bigger, due to underreporting and scarce epidemiological data.^7^ Besides the deaths, these accidents leave sequels to a substantial number of people.^8^

In Brazil, approximately 90% of snakebite envenomations are caused by pit vipers of *Bothrops* snakes.^6,9^ Two medically important species of the *Bothrops* genus are *Bothrops atrox*, which is widely distributed in tropical countries of South America east of the Andes, including the northern half of Brazil,^10^ and *Bothrops leucurus*, which inhabits the Atlantic Forest in northeastern Brazil.^6^ The only treatment for snakebite accidents is antivenom therapy, which markedly reduces the mortality rate, although it presents severe adverse effects, including anaphylaxis and serum sickness.^11^ There are also availability problems, as most accidents occur far from the urban centers, where the access to antivenom in health facilities is more difficult. Another limitation of the antivenom is its ineffectiveness in reducing local tissue damages, leaving permanent physical disabilities in a great proportion of patients.^12^ Therefore, there is an urgent need for the development of auxiliary therapeutic tools.^13^ SVMPs are the predominant protein (toxic) components in viperid venoms averaging around 33% of total venom proteins, particularly within the *Bothrops* genus (average of 45%).^14^ Although present in lower amounts, they are also found in the Elapidae family (around 5% on average)^14^ and in the Colubridae family (although venom composition data is limited for this group).^15^ Given their significant role in inducing both local and systemic toxicological reactions, they represent interesting targets for drug development.^16,17^

SVMPs are zinc-dependent proteases with a large structural similarity in the zinc-binding domain, mostly in the catalytic site, and are the main responsible of the local inflammation, hemorrhage and tissue necrosis, as well as in systemic hemotoxicity,^3,5,9,18,19^ and facilitating the diffusion of other toxins into circulation.^12^ They belong to the metzincins clan, a group of zinc-dependent metalloendopeptidases that includes other enzymes of medical relevance, such as ADAMs (A Disintegrin And Metalloproteinases) and the MMPs (Matrix Metalloproteinases), all of them presenting similar functions and structures, especially in the active site.^20^ The first drug candidates that target these enzymes combined a zinc-binding group (ZBG), mostly with a hydroxamate group, with a peptidomimetic structure, producing potent broad-spectrum inhibitors. Among them, the compounds batimastat (BAT) and marimastat (MAR)^21^ have a high affinity for these enzymes and have been cocrystallized with metalloproteinases, providing valuable structural information, especially about zinc/hydroxamate interactions.^22,23^ After that, a second generation of compounds was developed, including scaffolds that maximize hydrophobic interactions in the S1′ pocket, as the sulfonylated amino acid hydroxamates,^21^*e*.*g*. prinomastat (PRI) and CP471474 (CP4). The compounds PRI, BAT, and MAR achieved phase III in clinical trials,^23,24^ and showed inhibitory activity against other SVMP.^25^ MAR has also been shown to interact with toxins from the *Crotalus atrox* venom.^26^

**Figure 1:**
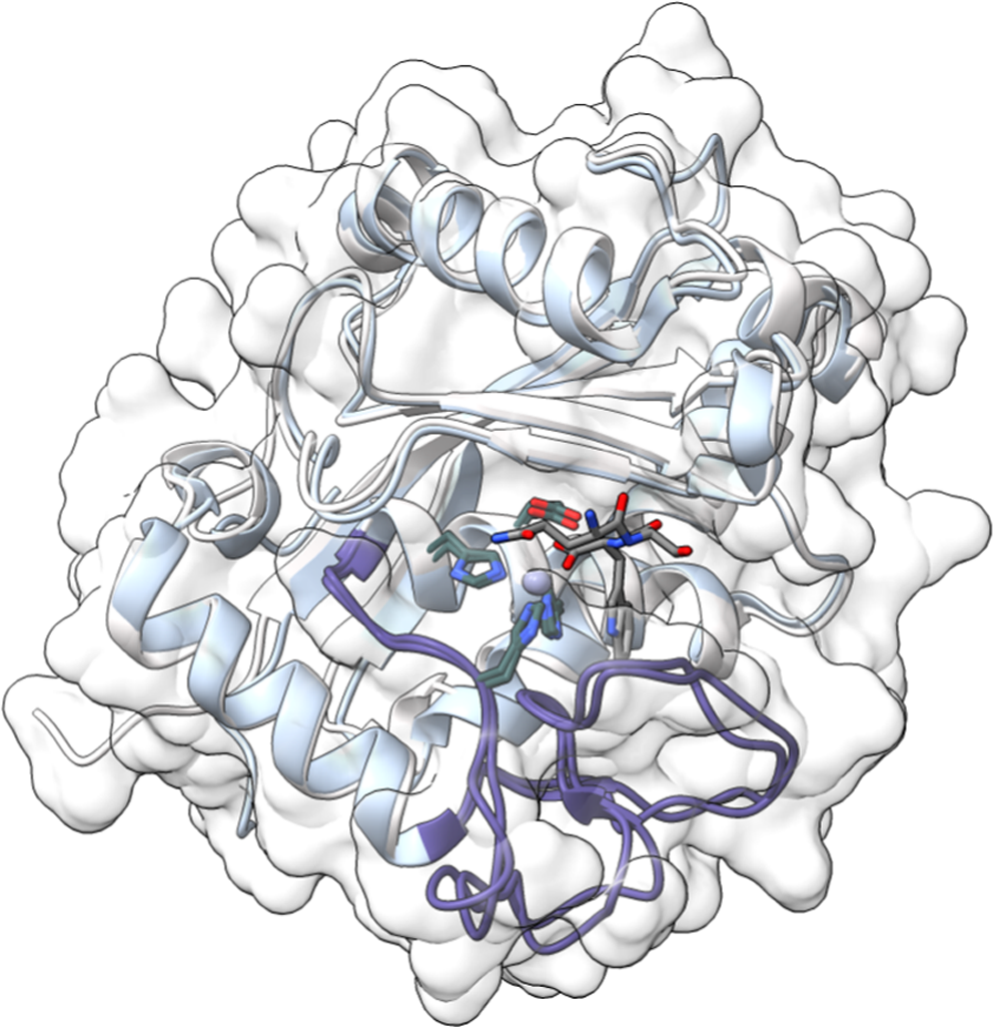
Superposition of the initial structures of the toxins analyzed in this study, the Atr-I model and the Leuc-a structure complexed with an endogenous inhibitor (PDB code: 4Q1L), displaying their secondary structures as ribbons and the molecular surface. In both toxins, the entire protein corresponds to the catalytic domain, which is also present in SVMPs PII and PIII, though these classes contain additional domains. Key amino acids of the catalytic site—His142, Glu143, His146, and His152—are highlighted in stick representation with carbon atoms in dark green, along with the tripeptide inhibitor QSW, with carbon atoms in gray, featuring the tryptophan side chain positioned in the S1′ hydrophobic pocket. The Ω loop is emphasized in purple for visual clarity, and the zinc atom is represented as a gray sphere.

The catalytic domain of the SVMPs presents an ellipsoidal shape with an active-site cleft that separates the N-terminal part of the molecule, composed of a highly twisted five-stranded β-sheet and four α-helices, from an irregularly folded C-terminal part^27,28^ (Figure 1). Within the active site, a loop commonly labeled as the Ω-loop in other metzincins possesses an important role in substrate recognition.^29^ The catalytic zinc ion is located at the bottom of the active-site cleft, coordinated with the three conserved histidines through their Nε2 atoms, and a water molecule that works as a nucleophile. This molecule is also anchored to a conserved glutamic acid residue, essential for catalysis^29^ by activating the nucleophilic water. Located adjacently to the zinc atom is the hydrophobic S_1_′ pocket, which plays a key role in substrate affinity.^30^

Despite the high structural similarity in the SVMPs catalytic domains, there are large variations in their hemorrhagic effect, including some that lack this activity.^5,19,31−33^ No direct correlation has been found between the amino acid sequences of these toxins and their hemorrhagic effects, suggesting that these differences may be attributed to structural characteristics, such as variations in electrostatic surface properties^28,34^ or the mobility of specific regions.^33^ Understanding these differences could be important for discovering effective blockers and developing biotechnological applications of nonhemorrhagic SVMPs, which have already been used clinically and others in preclinical and clinical studies for thrombosis treatment.^35−40^

Molecular modeling methods are widely used in structure-based drug design, reducing the time and high cost involved in this process. A common strategy involves virtual screening to identify target protein inhibitors, followed by more precise free energy calculations on a smaller set of compounds, and subsequent *in vitro* assays with the most promising candidates.^41,42^ These calculations can be performed using end-point methods such as Molecular Mechanics Poisson–Boltzmann Surface Area (MM/PBSA), or the Molecular Mechanics Generalized Born Surface Area (MM/GBSA) variation,^43,44^ or alchemical transformation methods like Thermodynamic Integration (TI) and Free Energy Perturbation (FEP).^45,46^

FEP has gained popularity in recent years due to technological advancements, enabling the evaluation of relative binding affinity for numerous potential drug candidates and facilitating optimization stages.^45−50^ However, while FEP is generally considered more accurate than MM/PB(GB)SA, it requires greater computational resources and more human intervention, despite recent advantages.^45,51−55^ In contrast, MM/PB(GB)SA are computationally more affordable, allowing for quick screening of large ligand libraries, and providing a good balance between precision and computational cost.^56,57^ These characteristics make MM/PB(GB)SA methods widely used in drug discovery and valuable for refining virtual screening results.^57−61^

MM/PB(GB)SA are commonly used to compute the enthalpic contribution of free energy of binding, using Poisson–Boltzmann equations (PB) or the Generalized Born (GB)^62^ method to account for the electrostatic part of the implicit solvation model. These methods use a continuous solvent model and estimate the free energy only in the initial and final state (*i*.*e.*, end-point methods), usually based on representative poses produced by molecular dynamics (MD) simulations. However, conventional force fields do not consider the atomic charge transfer and polarization effects, which are considerable limitations for modeling biological molecules containing metal atoms. In principle, these limitations could be overcome using quantum mechanical (QM) methods, although they present high computational costs that hamper their applications in modeling protein-containing systems. One standard solution in these situations is to use hybrid QM/MM methods,^63^ modeling a small region of the system with QM methods, usually the active site, and the rest of the system with MM, generally producing more precise results when compared with the classical method, especially for metalloenzymes.^64−66^ More specifically, we employ in this work two variants of the self-consistent charge density functional tight-binding (SCC-DFTB) methods, which are computationally efficient semiempirical QM (SQM) methods that have been intensively used in the study of zinc metalloenzymes.^67^ In addition, we test another commonly used semiempirical method, PM6, which can produce results for typical organic molecules comparable to those of *ab initio* QM methods.^68^

A challenging problem in the estimation of absolute or relative binding affinities is the calculation of the entropic contributions, mainly due to the requirement of exhaustively sampling the conformational, rotational, and translational motions of the host and ligand molecules both in complex and in their isolated states.^69^ Usually entropy estimations from MD simulations focus on the solute contributions to the binding free energy and are typically evaluated by applying normal modes analysis,^70^ which can be complemented with conformational entropy calculations.

**Figure 2:**
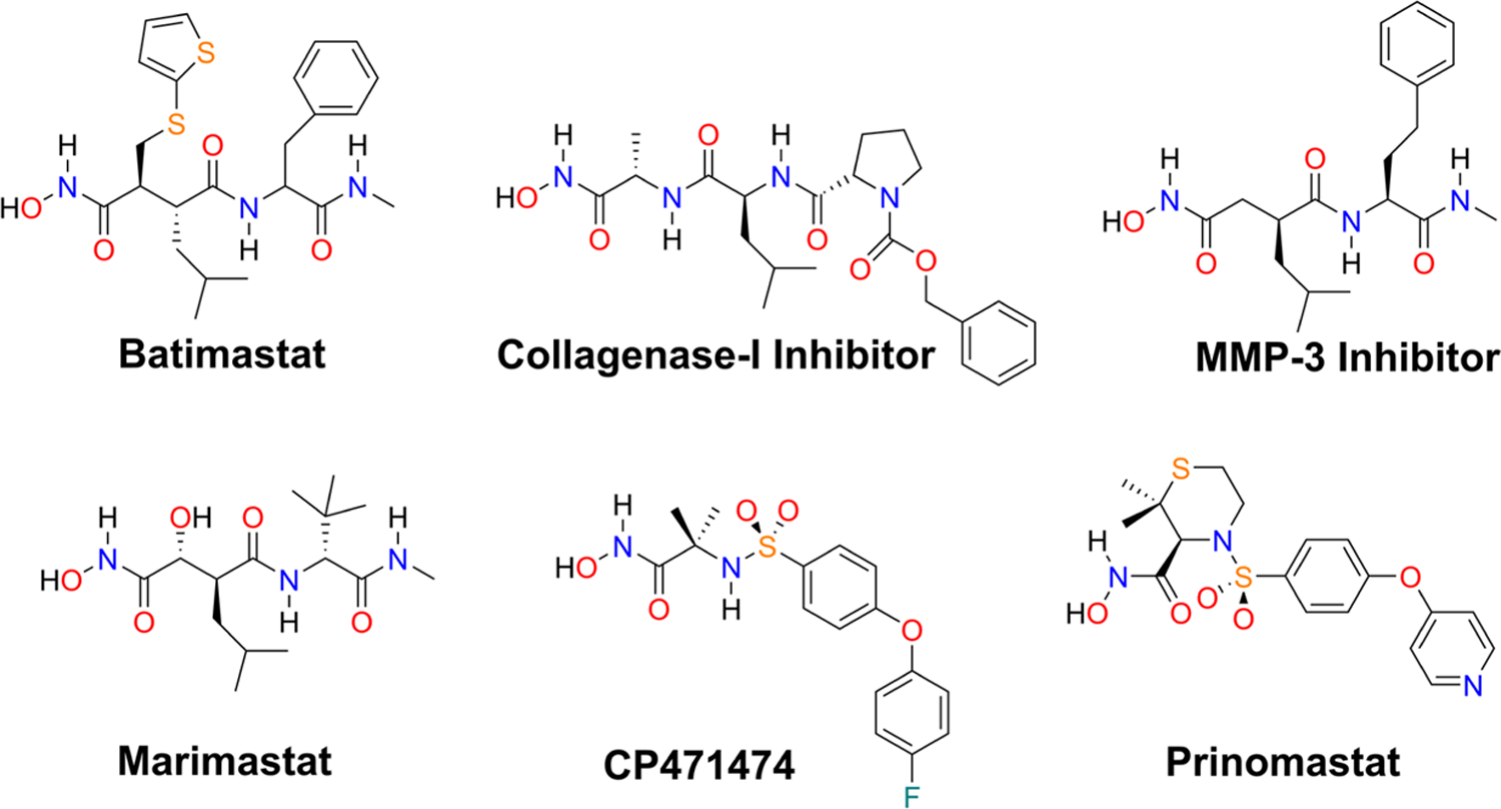
Broad-spectrum hydroxamate inhibitors evaluated in this study.

In this study, we have evaluated the inhibitory effect of six broad-spectrum metalloprotease inhibitors (Figure 2) against both Atroxlysin-I (Atr-I, hemorrhagic) and Leucurolysin-a (Leuc-a, nonhemorrhagic) proteinases. After confirming their blocking effect *in vitro*, we obtained the initial models of the toxin/ligand complexes by intensive docking calculations. After scoring the docking poses with QM/MM methods, we performed extensive MD simulations of the most likely structures in explicit solvent. For several inhibitor molecules, the simulations allowed us to examine various binding modes and/or charge configurations. The relative affinity of the toxin/ligand complexes was studied using a large variety of MM/PBSA-like scoring functions. Furthermore, different parameters and methods were used to compute the energetic and entropic terms that are combined to produce the scoring functions. These calculations also allowed us to determine the relative stability of the various binding modes and ligand charge configurations. The computational results were then compared with experimental data to select the scoring functions that present the best correlation results.

## Methods

### Enzymatic Assays

The enzymes Atr-I and Leuc-a were purified as previously described.^6,71^ Their enzymatic activity was first measured using 2 nM of each proteinase and concentrations ranging from 30 to 250 μM of the fluorescence resonance energy transfer (FRET)-peptide Abz-LVEALYQ-EDDnp, which was designed based on one of the substrate proteins of Atr-I.^72^ Assays were performed at room temperature with the buffer Tris–HCl 50 mM, CaCl_2_ 1 mM, pH 7.4, 2 nM of the toxins, 65 μM of the substrate, and concentrations of the selected inhibitors ranging from 0.1 nM to 75 μM. These assays with fluorogenic peptides were carried out for 1 min, with the hydrolysis being continuously monitored in a Synergy Biotek 2 fluorimeter, measuring the fluorescence at *k*_em_ = 440 nm and *k*_ex_ = 340 nm, following a procedure previously described.^72^ IC_50_, confidence intervals, and the other kinetic parameters were calculated by nonlinear regression analyses of substrate hydrolysis velocities with the software GraphPad Prim 6.0.^73^*K_i_* and IC_50_ values, for competitive inhibition, are related by Cheng Prussoff equation^74^ (eq 1). Efforts to determine the *K*_m_ values were hindered by limited solubility of the substrate, preventing reaching the maximum reaction rate. As a result, the *K*_m_ calculation was not achievable and it was concluded that the substrate concentration used in the test was considerably lower than the actual *K*_m_ values. Therefore, the IC_50_ values from the kinetic tests were considered a reasonable approximation of the *K*_*i*_ values.

The compounds batimastat (BAT), CP471474 (CP4), marimastat (MAR), and prinomastat (PRI) were bought from Sigma-Aldrich, and collagenase-I inhibitor (COL) and mmp-III inhibitor (MMP) were bought from MerckMillipore.
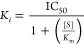
1

### Isothermal Titration Calorimetry Assays

Purified Atr-I was dialyzed at 4 °C against a large excess of 1 mM CaCl_2_ mM, lyophilized, and then dissolved in the same buffer used in enzymatic assays. Calorimetric experiments were conducted, at 25 °C, in an ultrasensitivity VP-ITC instrument (MicroCal Inc., Northampton, MA). The buffer solution was degassed before use. The inhibitors were first dissolved in dimethyl sulfoxide (DMSO), and then diluted in the same buffer to a concentration of 0.5 mM. Protein and inhibitor solutions were filtered before ITC experiments. The sample cell was filled with the protein solution and the reference cell with buffer. After an initial preinjection of 1 μL, aliquots of 10 μL of the inhibitor solution were stepwise injected at 5 min intervals, into the sample cell containing a protein solution in the range of 23.1–34 μM, until complete enzyme saturation. Blank titrations of ligand into buffer alone were also performed to correct for heat generated by dilution and mixing. Data were analyzed using an equal and independent sites model (noncooperative model) and the ITC module of Origin 7.0.

### Computational Methodology

**Scheme 1:**
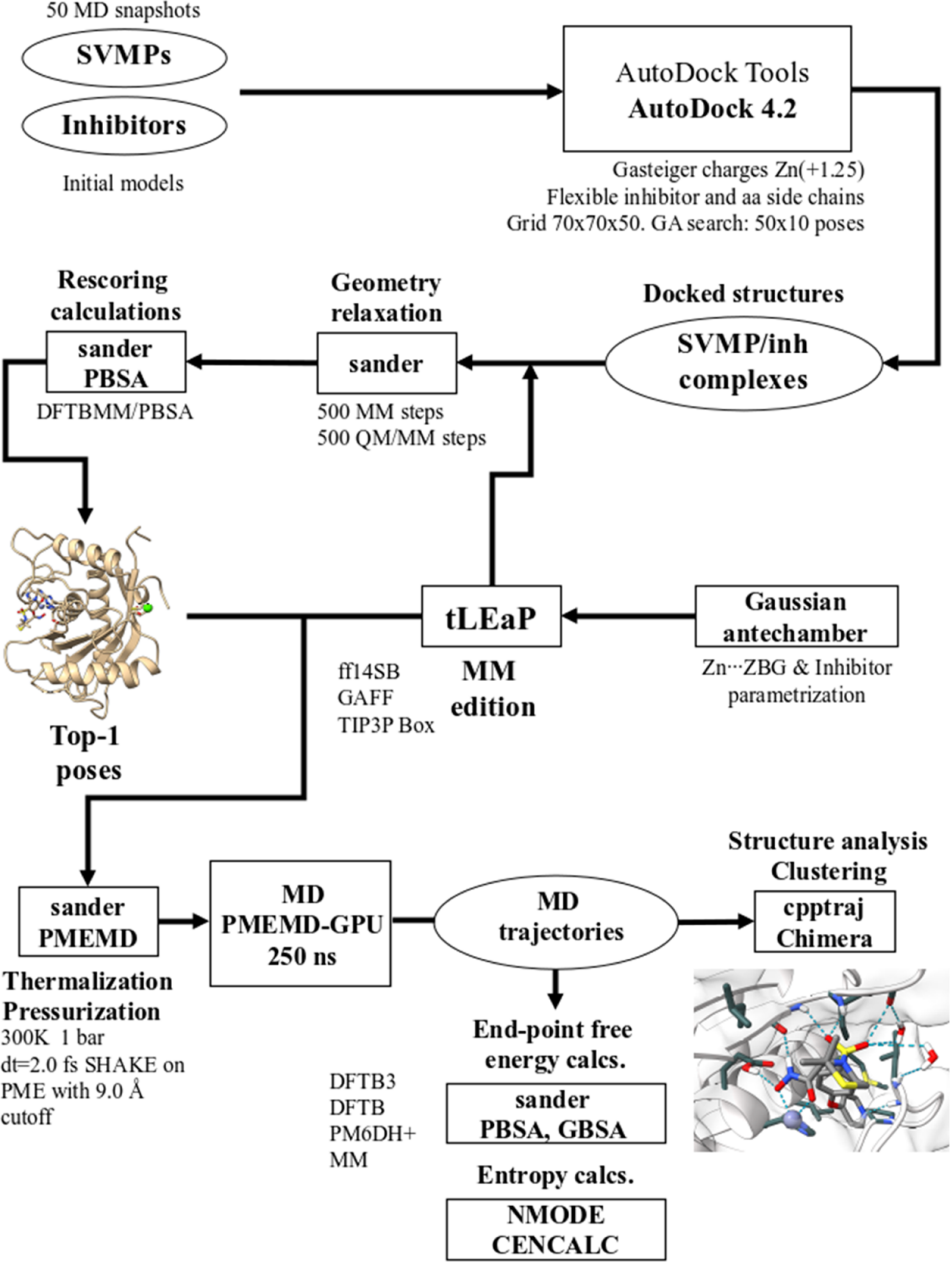
Computational Workflow Used on This Work

The general scheme of the computational methodology used in this work is summarized in Scheme 1.

### Setup of the Docking Calculations

#### Initial Structures

To introduce protein flexibility into the docking procedure, we selected equally spaced 50 snapshots from our previous 250 ns MD simulation of Leuc-a in complex with an endogenous tripeptide,^28^ which was carried out in explicit solvent starting from the X-ray PDB ID: 4Q1L structure.^18^ Similarly, for the Atr-I enzyme, we extracted 50 MD snapshots from the corresponding MD simulation starting from a comparative model previously reported.^75^ These structures contain two metal ions, the Zn^2+^ ion, coordinated to the Nε2 atoms of His_142_, His_146_, and His_152_, and the Ca^2+^ ion, which exhibits octahedral coordination with the side chains of Glu_9_, Asp_93_, Asn_200_, the carbonyl backbone of Cys_197_ and two water molecules in Leuc-a. These ligands are the same in Atr-I, except for the Glu_9_, substituted by Asp_9_. All the MD snapshots were postprocessed by removing the coordinates of the tripeptide, while water molecules and the Zn^2+^ and Ca^2+^ metal ions were maintained. Initial coordinates for the heavy atoms of the BAT inhibitor were taken from the crystallographic structure of the MMP-12 protein in complex with BAT (PDB code: 1JK3). For MAR, we extracted the inhibitor coordinates from the ADAM 33/MAR complex (PDB code: 1R55). Subsequently, H atoms were added to the structures using the Avogadro software.^76^ The initial structures of remaining inhibitors were prepared by modifying the batimastat or marimastat molecules with the building tools of Avogadro.

#### AutoDock Calculations

To dock each inhibitor within the catalytic site of the SVPMs, we employed AutoDock 4.0,^77^ which uses a Monte Carlo simulated annealing technique for configurational exploration of protein–ligand complexes with a rapid energy evaluation using grid-based molecular interaction potentials built from van der Waals (vdW), electrostatic and desolvation contributions. The AutoDock scoring function that estimates several binding free energy contributions (electrostatics (based on Gasteiger charges), vdW, and H-bond interactions, as well as desolvation) was used.^78^ We modified the atomic charges of the Zn^2+^ ion and its ligands to promote good coordination between the ZBG (*i*.*e*., hydroxamate) and the zinc ion, following a strategy similar to those reported in former works.^79,80^ Thus, we used a fractional charge for Zn (+1.25) that corresponds to the electrostatically fitted atomic charge of the Zn ion in a small cluster model, [Zn(methyl-imidazole)_3_]^2+^, as calculated at the B3LYP/6-31G(*p*) level of theory using the *Gaussian09* program.^81^ To preserve charge integrity, a +0.75 charge was distributed evenly throughout the Zn-coordinated His_142_, His_146_, and His_152_ residues (see the resulting charges in Figure S1). The Glu_143_ side chain was neutralized by adding a hydrogen atom and selecting the proper Gasteiger charges for the carboxylic group. Similarly, the hydroxamic acid of the SVMP ligands was modeled in its anionic form adopting the charges shown in Figure S1. We note that this mode of binding aligns with the p*K*_a_ shift of +2.9 units that have been calculated for the Glu_143_ residue of the BaP1 SVMP^75^ and agrees with the commonly assumed mode of binding of MMP and ADAM inhibitors bearing hydroxamic and carboxylic acids as zinc-binding groups (ZBGs).^82,83^

To explore multiple binding options, AutoDock requires precalculated grid maps for the receptor, in which information on electrostatics, H-bond and steric constraints are stored for each atom type in the ligand. We centered the grid maps on the catalytic Zn atom of the SVMPs and defined 70 × 70 × 50 grid points along each Cartesian axis with a spacing of 0.375 Å. To sample the toxin/ligand conformational space, the SVMPs atoms were kept rigid. We employed the genetic search algorithm as implemented in AutoDock to perform 50 rounds of docking starting from different random seeds, generating 10 poses each, and resulting in a total of 500 docking poses for each SVMP/ligand complex.

#### Rescoring of the Docking Poses

To better discriminate among the docking poses, we relaxed the enzyme···ligand interactions employing MM & QM/MM geometry optimizations that improve particularly the representation of the Zn···ligand contacts. The geometry optimizations were followed by single-point QM/MM calculations and electrostatic Poisson–Boltzmann calculations to rescore the docking poses. In these calculations, the systems were represented with the ff14SB version of the all-atom Amber force field.^84^ The Zn^2+^ and Ca^2+^ ions were represented by the nonbonding parameters developed by Li et al.^85^ For the ligands, we generated their MM representation using the Generalized Amber Force Field (GAFF) with electrostatically derived atomic charges at the B3LYP/6-31G(d) level. This parametrization task was done automatically using the *antechamber* tools available in the AmberTools14 package^86^ coupled with the *Gaussian09* program.^81^ QM/MM calculations were performed at the semiempirical SCC-DFTB level^67,87^ with extended parameters for Zn.^88^ The QM region comprised the His_142_ and Glu_143_ residues capped by Ace and Nme moieties, respectively, plus the side chains of His_146_ and His_152_ (capped by H-link atoms at the Cα atom), the Zn^2+^ ion and the hydroxamate group of the inhibitor molecules, *i.e.*, the group H–C=O–NH–O, where the H atom attached to C is the H-link atom (Figure S2). The decision to include the entire His_142_ and Glu_143_ residues in the QM region, rather than just their side chains, was made because they are consecutive residues. Leaving the His_142_-Gly_143_ bond in the MM region would have resulted in QM-MM linkages being too close to each other, which could lead to overpolarization effects.

Each docking pose was relaxed by moving the cluster model of the active site, the inhibitor molecule, and the nearby SVMP residues (105–111, 166–171, 138–139, and 176) during 500 conjugate-gradient cycles of MM minimization followed by 500 steps of QM/MM minimization using the *sander* program with a distance-dependent dielectric (ε = 4*r*) to mimic solvent screening and with a 20.0 Å cutoff for nonbonded interactions. Subsequently, single-point QM/MM calculations in the gas-phase and with no cutoff were performed for the SVMP/inhibitor complex, the SVMP receptor, and the isolated inhibitor molecule using the optimized geometry of the SVMP/inhibitor complex. Similarly, the solvation energy of the complex and the separate fragments was evaluated with the *pbsa* program included in AmberTools.^86^ In this case, the electrostatic potential was calculated by solving the nonlinear Poisson–Boltzmann equation and taking the atomic charges and radii from the ff14SB representation, except for those atoms located in the QM region during the previous QM/MM calculations, for which the SCC-DFTB Mulliken charges were used instead, following recommendations from AMBER developers.^89^ The total charge of the H-link atoms was distributed evenly among the remaining QM atoms. The nonlinear PB equation^90^ was solved using identical settings to those adopted for the MM/PBSA calculations (see below).

The score of the docking poses was calculated using a more robust physics-based energy function. Initial scoring combined the SCC-DFTB energy and the PBSA solvation energy of the *i*-th pose from the *j*-th MD frame, that is,
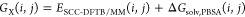
2where X = SVMP/inhibitor, SVMP, or inhibitor. Then each pose denoted by the (*i*, *j*) pair was rescored using the following expression:
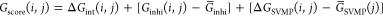
3where Δ*G*_int_ is the SVMP/inhibitor interaction energy and the two terms in brackets estimate the distortion energy contribution to the relative stability of the poses due to changes in the internal geometry of the inhibitor and SVMP fragments. For assessing the distortion effects, we obtained the average energy of each inhibitor (*G̅*_inhi_) in the full set of poses, but the corresponding term for the SVMP system (*G̅*_SVMP_(*j*)) was just the average value over the docking poses for a given MD snapshot *j*.

#### Setup of the MD Models for the Atr-I and Leuc-a Systems Complexed with the Hydroxamate Inhibitors

The best-ranked docked poses were selected as starting coordinates for the Atr-I and Leuc-a toxins complexed with the inhibitors. The inhibitor binding modes are characterized by the coordination of the hydroxamate group with the zinc ion, as observed in the crystallographic structures with metalloproteases and this group of inhibitors,^22,91,92^ the positioning of a nonpolar group within the S_1_′ pocket, and the interaction between the hydroxyl oxygen atom of the hydroxamate group with the Glu_143_ side chain. Hydrogen atoms were added to the toxins models by the LEaP program included in the AMBER14 package^86^ and the ff14SB version of the all-atom Amber force field^84^ was used to represent the SVMP toxins, while the GAFF^93^ was used for the inhibitors, except for the parameters derived for the zinc environment (as already described). The calcium ion was modeled by the nonbonding parameters developed by Li et al.^85^

The charge configurations of the Zn-bound hydroxamate and the Glu_143_ side chain (Figure S2) were selected based on our previous MD simulations of these same toxins complexed with an endogenous peptide inhibitor,^28^ and other studies with the structurally similar MMP complexed with inhibitors bearing zinc-binding groups (ZBGs), that presented inhibitor binding modes consistent with a negatively charged ZBG and a neutral carboxylic group for the conserved Glu side chain.^82,83,94^ Therefore, this configuration was assumed for all toxin/inhibitor MD simulations.

The proper coordination environment around the Zn ion is preserved during the extended MD simulations through the use of a bonded MM representation, in which the metal ion is linked to the His_142_, His_146_, and His_152_ Nε2 atoms and to the hydroxamate group oxygen atoms of the inhibitors molecules. After QM geometry optimization, the reference geometry (bond lengths and angles) was obtained from a cluster model (Figure S2). This optimization was performed at the B3LYP/6-31G(d) level of theory^95−97^ combined with the PCM continuum solvent model^98^ (ε = 20.0), as implemented in the *Gaussian09* program.^81^ From the reference geometry, a set of perturbed structures was built by modifying gradually and selectively the bond distances (±0.025, ±0.05, ±0.075 Å) and angles (±3, ±6, ±9°) involving the Zn atom, leaving the internal geometry of the metal ligands unaltered. After computing the B3LYP-PCM energies of the perturbed geometries, the force constants for the MM bonds and angles were obtained by fitting a second-order polynomial to the relative energies. All the torsions associated with the Zn-ligand interactions were set to zero.

For each of the modes of binding between a hydroxamate inhibitor and an SVMP derived from the Autodock calculations, we obtained a set of specific atomic charges representing the Zn ion, the three imidazole ligands, the side chain of the Glu_143_ side chain and the full inhibitor molecule. To this end, the atoms in this Zn environment were assigned to a QM region, and the docked SVMP/inhibitor complexes were partially relaxed using the QM/MM interface implemented in *sander* in combination with *Gaussian09*. The QM region was described at the B3LYP/6-31+G* level of theory, while the rest of the protein atoms were treated with the ff14SB force field with no cutoff. H-link atoms were inserted by *sander* at the corresponding Cα-Cβ bonds. During the QM/MM geometry optimization, only the QM atoms and the side chains of the closest residues were allowed to move until the root-mean-square of the Cartesian elements of the gradient was less than 0.02 kcal/(mol Å) (2 × 10^–5^ in au). These QM/MM calculations further confirmed the stability of the Glu_143_-COOH···hydroxamate/carboxylate contacts. For each QM/MM structure, we extracted the coordinates of the QM region and performed a single-point B3LYP/6-31G(d) calculation using *Gaussian09*. Then, we derived atomic partial charges fitted to the B3LYP/6-31G(d) PCM (ε = 20) electrostatic potential using the RESP methodology. During the RESP fitting procedure, we assigned a zero value to the atomic charges of the H-link atoms. To preserve the integral charge of the whole system, the partial charges of the backbone atoms of the residues bound to Zn were slightly modified. Finally, the Lennard-Jones parameters for the Zn ion were those of Li et al.,^85^ and other parameters and atom types were taken from the ff14SB force field for the protein atoms and the GAFF force field for the inhibitor atoms. The files of the QM/MM optimized geometries of the Zn cluster with the inhibitors, or the isolated inhibitors, along with all the derived charges, have been uploaded to Zenodo.

#### MD Simulations in Explicit Solvent

The initial Leuc-a and Atr-I structures were placed in a rectangular box of TIP3P^99^ water molecules that extended 18 Å from the toxin atoms. For the Leuc-a system, 4 Cl^–^ counterions were required to neutralize the system and were described using the TIP3P-water-model-specific ion parameters.^100^ Only 1 Na^+^ counterion was required to neutralize the Atr-I system. The settings of the MD simulations were identical to those used in our previous simulations.^28^ Briefly, periodic boundary conditions were applied, and long-range interactions were described by the Particle–Mesh–Ewald method. Solvent molecules and counterions were initially relaxed using energy minimizations with the *sander* program. Then the full systems were minimized and heated gradually to 300 K at constant NVT conditions. Subsequently, the systems were pressurized by running a constant NPT simulation with a Monte Carlo barostat controlling the pressure. The production phase of the simulations comprised 250 ns, which were run at constant NVT conditions using the accelerated version of the *pmemd* code for Graphical Processing Units.^101,102^ Coordinates were saved for analysis every 2 ps. During the MD simulations, Langevin dynamics^103^ was employed to control the temperature and the length of all R–H bonds was constrained with the SHAKE algorithm.^104^

Root-mean squared deviations (RMSD) calculations with respect to the first structures and cluster analyses were performed using the *cpptraj*(105) module of Amber14. The clustering analyses employed a hierarchical agglomerative (bottom-up) approach, with average linkage, an epsilon value of 1.25, and RMSD values for specific amino acids interacting with the inhibitors for over 20% of the time during the MD simulations. For both toxins, the selected amino acids included Thr_107_, Ile_108_, Gly_109_, Ile_110_, Ala_111_, His_129_, His_142_, Glu_143_, His_146_, His_152_, and Leu_170_. For Atr-I, Pro_106_, Val_113_, Met_135_, Ile_138_, Pro_168_, Val_169_, Ser_171_, and Pro_174_ were also considered. For Leuc-a, additional amino acids were Val_102_, Glu_105_, Glu_106_, Met_127_, Val_138_, Ile_165_, Ala_167_, Asp_168_, Thr_169_, and Phe_176_.

H-bond and vdW contacts between the SVMP enzyme and the ligands were analyzed using a FORTRAN code developed locally. In these analyses, the H-bond interactions were only characterized based on geometrical criteria (*e*.*g*., X···Y distance < 3.5 Å and X–H···Y angle > 120°), and the nonpolar interactions were scored by evaluating an empirical dispersion attraction term^106^ between pairs of atoms belonging to different hydrophobic groups. The criteria for assessing the occurrence of dispersion interactions between two groups were: (a) the total pairwise dispersion energy is larger than 0.5 kcal/mol in absolute value; (b) the distance between the centers of mass of the two interacting groups is below 12.0 Å.

#### End-Point Free Energy Calculations

The end-point MM-PBSA method^44,57^ was used to estimate the interaction energy between the toxins and the inhibitor molecules using the coordinates derived from the MD simulations. In addition to the standard MM-PBSA protocol, QM/MM-PBSA variants^63^ using the SCC-DFTB,^87^ SCC-DFTB3,^107^ and PM6DH+^68^ Hamiltonians for the QM region were also considered. Two distinct QM regions were tested in the calculations with SCC-DFTB: one utilizing the same QM region as in the docking poses rescoring, and another encompassing the whole inhibitors in the QM region, rather than just the hydroxamate group. For all these variations, the GBSA solvation model^108^ was also evaluated.

The MM/PBSA energy of a solute molecule is obtained as
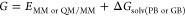
4where *E*_MM or QM/MM_ is the gas-phase energy of a solute molecule, including the 3RT contribution due to the translational and rotational degrees of freedom, and Δ*G*_solv_ is its solvation energy in aqueous solution.

The *E*_MM_ terms were computed with the *sander* program without explicit bonds between the catalytic Zn^2+^ ion and the inhibitors. In these calculations, the Zn^2+^ ion was described by nonbonding parameters of Li et al.^85^ that reproduce experimental ion-oxygen distance values and coordination numbers of the first solvation shell. The solvation energy is composed of one term that estimates the electrostatic solvation energy, obtained by Generalized Born (GB) or Poisson–Boltzmann (PB)^109^ methods, complemented by the dispersion and cavitation interactions for the nonpolar solvation term, as implemented in the *pbsa* program included in the AMBER 14 suite.^110^ The toxins were represented by the ff14SB force field,^84^ with additional parameters for Zn^2+^ and Ca^2+^ ions,^85^ and the inhibitors by the GAFF force field, except for atomic charges, that were substituted by the RESP charges computed as described earlier. The nonlinear PB equation^90^ was solved with a space grid of 0.33 Å and the dielectric boundary was the contact surface between the radii of the solute and the radius (1.4 Å) of a water probe molecule.

The interaction energy Δ*G*_int_ between the SVMP enzyme and the inhibitors is computed with the formula
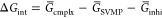
5where *G̅*_complx_, *G̅*_SVMP_, and *G̅*_inhi_ are the average MM/PBSA energies of the SVMP/inhibitor complex, the SVMP and the inhibitor, respectively, which were derived from calculations done on 2500 MD snapshots extracted in regular intervals of 100 ps during the MD simulation of the complex, after the water molecules and counterions were striped-off. Thus, the *G* energies of the SVMP and the inhibitor were computed using the coordinates retrieved from the MD trajectory of the complex. This approach, which minimizes statistical uncertainty, is also known as the one-trajectory approximation^44^ and assumes that the relative binding energy of small ligands can be approximated by the interaction energies.

For relatively large ligand molecules, the free energy cost associated with the reorganization of the ligand may influence the binding free energy. In the context of the MM/PBSA methods, the distortion energy of the bound inhibitors is readily obtained by subtracting their average MM/PBSA energies in the bound state (*G̅*_inhi_) and in the free state *G̅*_inhi_^free^ described by an independent MD simulation, that is, Δ*G*_dis_ = *G̅*_inhi_ – *G̅*_inhi_^free^. The role played by the distortion of the SVMP enzyme was similarly analyzed and the corresponding structures of the isolated SVMPs were retrieved from our previous MD simulation of the Atr-I and Leuc-a enzymes.^28^

The relative stability of the SVMPs/inhibitor complexes was also assessed using semiempirical QM/MM calculations that account for electronic effects like polarization and charge transfer within the selected QM region. In particular, we employed the DFTB^87^ and DFTB3^107^ versions of the self-consistent charge density functional tight-binding (SCC-DFTB) method.^67^ The DFTB/DFTB3 Hamiltonians are expressed in terms of the so-called Slater–Koster parameters, whose values were selected from the mio/3OB sets,^111,112^ specifically optimized for biomolecular calculations. The DFTB3 energies were further complemented with the so-called D3H4 empirical corrections for a better description of dispersion and hydrogen-bonding interactions.^113^ Similarly, we performed QM/MM calculations with the semiempirical PM6 Hamiltonian enhanced with hydrogen-bonding corrections and standard dispersion energy (PM6DH+).^68^ All the QM/MM calculations were computed using the *sander* program except the empirical D3H4 corrections obtained with the *cuby4* framework.^114^ In addition, the gas-phase QM/MM energies were augmented with the solvation energy term computed with the PB or GB methods using the Mulliken charges for the QM atoms as explained above. Prior to the computation of the QM/MM-PB(GB)SA energies, the QM region was first relaxed using 50 optimization steps driven by the Truncated-Newton Conjugate Gradient (TNCG) method implemented in the *sander* program.

#### Entropy Calculations

Although entropy calculations considering all degrees of freedom of the SVMP/inhibitor complexes are extremely challenging due to the relatively large size of the systems,^115^ the estimation of the absolute or relative entropy corresponding to the inhibitor and enzyme molecules may complement the results of the MM/PBSA-like calculations.

Assuming that the potential energy surface of a biomolecule consists of a collection of disjoint harmonic wells,^115^ its absolute entropy *S* can be defined as
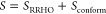
6where *S*_RRHO_ is the average entropy over the set of energy wells associated with the translational + rotational + vibrational DOFs (Degrees Of Freedom). Each *S*_RRHO_ value is obtained by MM normal mode calculations of the Hessian matrix and applying quantum mechanical formulas derived within the rigid rotor and harmonic oscillator (RRHO) approximations.^115^ On the other hand, *S*_conform_ is the conformational contribution associated with the population distribution {*P*_α_} of the different minima, that is,
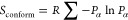
7

Due to their computational cost, the RRHO entropy calculations were performed on a subset of 250 frames equally spaced along the MD trajectories. Before the normal mode calculations, the energies of the systems were minimized until the RMSD of the elements in the gradient vector was less than 10^–6^ kcal/mol Å. To avoid extensive changes in the internal geometry, the systems contained the coordinates of the solute atoms and those of a buffer layer of water with a ∼6 Å thickness around the solute atoms.^116^ During the energy minimizations, the water molecules are kept fixed. Subsequently, the geometrical calculation of the Hessian matrix and the normal mode calculations were restricted to the active region comprising the solute atoms. The geometry optimizations were performed with the TCNG method algorithm available in the *sander* program while the normal mode calculations were performed using a locally modified version of the *nmode* program.

Conformational entropies (*S*_conform_) were calculated using the *cencalc* program,^117^ which selects first a set of rotatable dihedral angles using topology information. Using MD trajectory coordinates (125,000 frames in our case), *cencalc* discretizes the time evolution of the dihedral angles by representing first the continuous probability density function (PDF) of each dihedral angle by a von Mises kernel density estimator. By finding the maxima and minima of the PDF, the time series containing the values of the corresponding dihedral angle is transformed into an array of integer numbers labeling the accessible conformational states. The probability mass functions *P*_i_ of the individual dihedral angles are calculated and *S*_conform_ is estimated as the sum of the marginal (first-order) conformational entropy of each dihedral angle *i*:
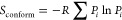
8

For computational reasons, the *S*_conform_ calculations were performed separately for the SVMPs and ligand molecules, both in their free forms in an aqueous solution and in their complexed forms, resulting in reasonably well-converged entropy values. The *S*_conform_ of the SVMPs was estimated considering a truncated model of comprising the active site region (see below).

Besides the RRHO calculations, we also estimated the change in the translational and rotational entropy of the inhibitor molecules using the statistical mechanics approximations introduced by Swanson et al.^118^ and later refined by Aqvist and co-workers.^119^ This approach allows the calculation of entropy contributions directly from MD simulations. Assuming that the motions of the center of mass of the inhibitor follow a Gaussian distribution, the translational entropy change upon inhibitor binding can be expressed as
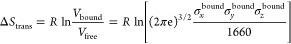
9where *V*_bound_ and *V*_free_ are the (translational) configurational volumes in the bound and free states of the inhibitor molecule. *V*_free_ is the reference volume determined by the standard concentration of 1 M (1660 Å^3^ per molecule) while *V*_bound_ is estimated by the product of the root-mean squared flexibility (RMSF) in the Cartesian directions of the inhibitor molecule along the MD simulation of the complexes. For calculations of rotational entropies, again assuming that motions have a Gaussian distribution, we used the approximation suggested by Carlsson and Aqvist,
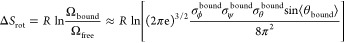
10where the rotational volume of the ligand, Ω_bound_, is expressed in terms of the RMSF in the three Euler angles describing its relative orientation with respect to the protein molecule in the bound state. The calculations of Δ*S*_trans_ and Δ*S*_rot_ were done using the *cpptraj* program and an in-house script developed in our laboratory.

#### Scoring Functions

Clearly, there are many variants of the MM/PBSA approach that arise from the choice of the MM (or QM) energy method, the solvation method, the entropy corrections, etc. so that these methods can be considered as physics-based scoring functions. In terms of ligand affinity rankings, these scorings may have enough predictive capacity despite their statistical and systematic errors in their estimation of binding free energies.^120^ The performance of a particular scoring is limited by the accuracy of the energy method and implicit solvent model, the potential enthalpy/entropy imbalances, etc., and, therefore, we tested in this work many MM(SQM)/PB(GB)SA scoring functions to determine their correlation with experimental affinities of the SVMPs/inhibitor complexes. All the tested scoring functions (*f*_score_^*E*/Δ*G*_solv_^), which include at least the mean value of the SVMP···inhibitor interaction energy term (Δ*G*_int_) as evaluated with the selected energy/solvation models (*E*/Δ*G*_solv_), can be augmented with the corresponding averages of the distortion terms (Δ*G*_dis_^inhi^ and/or Δ*G*_dis_^SVMP^) and/or entropy corrections (Δ*S*). Thus, the general expression of the *f*_score_^*E*/Δ*G*_solv_^ functions can be formally expressed as
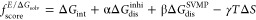
11where α, β, γ = 0/1 in order to switch on/off the associated free energy components. On one hand, the gas-phase energies (*E*) were computed with the nonbonding MM force field (IOD or HFE parameters for Zn^85^) or with one of the SQM/MM schemes with SQM = SCC-DFTB,^88^ SCC-DFTB3^107^ with D3H4 corrections or PM6 with DH+^113^ corrections. For brevity, these SQM/MM methods will be denoted hereafter as DFTB, DFTB3, and PM6. On the other hand, the PBSA and GBSA solvation models were considered for each energy method. Hence a total of 12 *E*/Δ*G*_solv_ combinations were tested.

We also investigated the impact of the settings of the (SQM/MM)/PB(GB)SA calculations on the performance of the scoring functions. For example, we considered two different QM regions for the DFTB method: (a) the QM_large_ region that comprises the catalytic [Zn(imidazole)_3_]^2+^ cluster, the Glu_143_-COOH side chain, and the full inhibitor molecules; (b) the QM_small_ one including [Zn(imidazole)_3_]^2+^, the Glu_143_-COOH side chain and only the hydroxamate group of the inhibitor molecule. In principle, the QM_small_ option combines a QM description of the important Zn···ZBG interactions with a MM representation of the H-bond, electrostatic, and vdW effects determining other enzyme···inhibitor interactions. The QM_large_ option may result in an unbalanced description of the specific H-bond/electrostatic interactions due to overpolarization of the relatively large QM region involving the inhibitor molecule (but not the surrounding enzyme residues). In addition, we examined the potential influence of the size of the systems. Hence, besides calculating the interaction and distortion free energy components on the full enzyme/inhibitor systems, we focused on the role played by the short-range interactions and the strain effects in the active site region by evaluating the Δ*G*_dis_ and Δ*G*_int_ energies on truncated SVMP/inhibitor subsystems. In addition, the calculations on smaller systems can benefit from lower statistical noise in the resulting average values. The truncated enzyme/inhibitor structures comprise the inhibitor molecule and all residues within ∼10 Å to the inhibitors′ atoms (Figure S3), which is formed by segments 101 to 112 (β-sheet), 123 to 128 (β-sheet), 133 to 148 (α-helix) and 148 to 177 (Ω loop). Terminal *N*-methylamine or acetyl groups were placed at the C and N backbone atoms of those residues cut out from the protein main chain by the truncation process.

Concerning the entropy Δ*S* terms to be added to the scoring functions, we examined four different alternatives that involved selected degrees of freedom and methodologies,
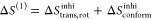
12
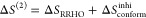
13
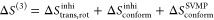
14
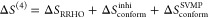
15

In this way, the entropy scorings Δ*S*^(1)^ and Δ*S*^(3)^ incorporate the change in translational and rotational entropy of the inhibitor as evaluated with the classical statistical mechanics approximations (eqs 12 and 14 ) with the conformational entropy estimations obtained with *cencalc*. Reciprocally, Δ*S*^(2)^ and Δ*S*^(4)^ add the full RRHO entropy of the toxin/inhibitor complexes with the conformational entropies (eqs 13 and 15).

To assess the performance of the scoring functions in predicting affinity rankings of the SVMP/inhibitor complexes, the MD-averaged values of each scoring function were used to compute the correlations with the experimentally based Δ*G* values using the Pearson correlation method with the R program,^121^ and the best results were selected based on the *p*-values. It must be stressed that whenever there was more than one binding mode for a toxin/inhibitor complex, the smallest value for each scoring function (i.e., the most favorable one) was selected for the correlation evaluation.

#### Structure Analysis

Structures and trajectories were visualized using Molden,^122^ Chemdraw14,^123^ and ChimeraX.^124^ The similarity coefficients of the inhibitors were calculated with OpenBabel.^125^

## Results and Discussion

### Characterization of SVMP Inhibitors

**Figure 3:**
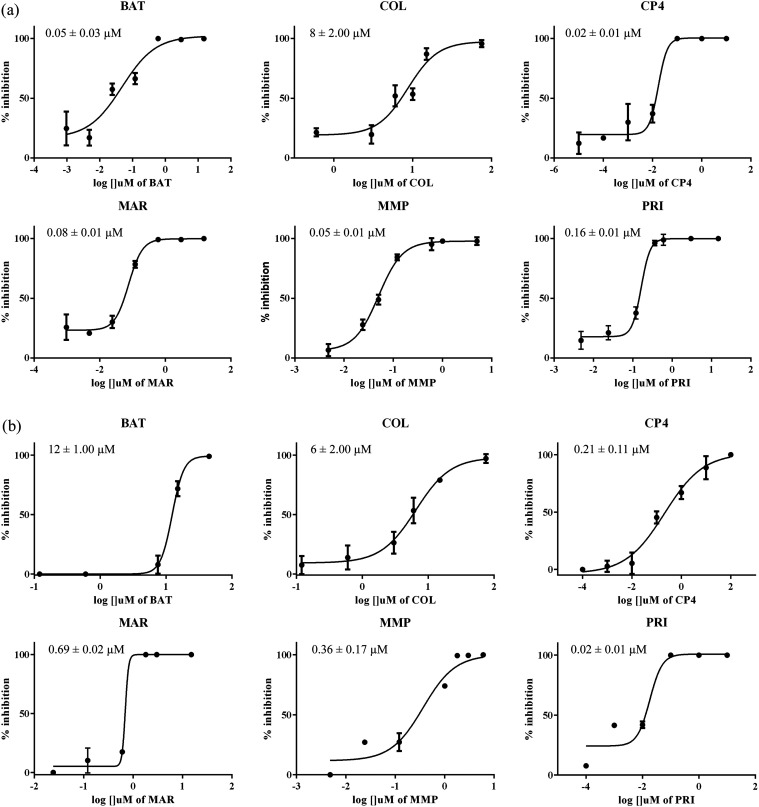
IC_50_ curves and values (μM) obtained by the enzymatic kinetics assays using broad-spectrum inhibitors and (a) Atr-I or (b) Leuc-a. Each curve was determined based on five to seven compound concentrations, in triplicates.

**Figure 4:**
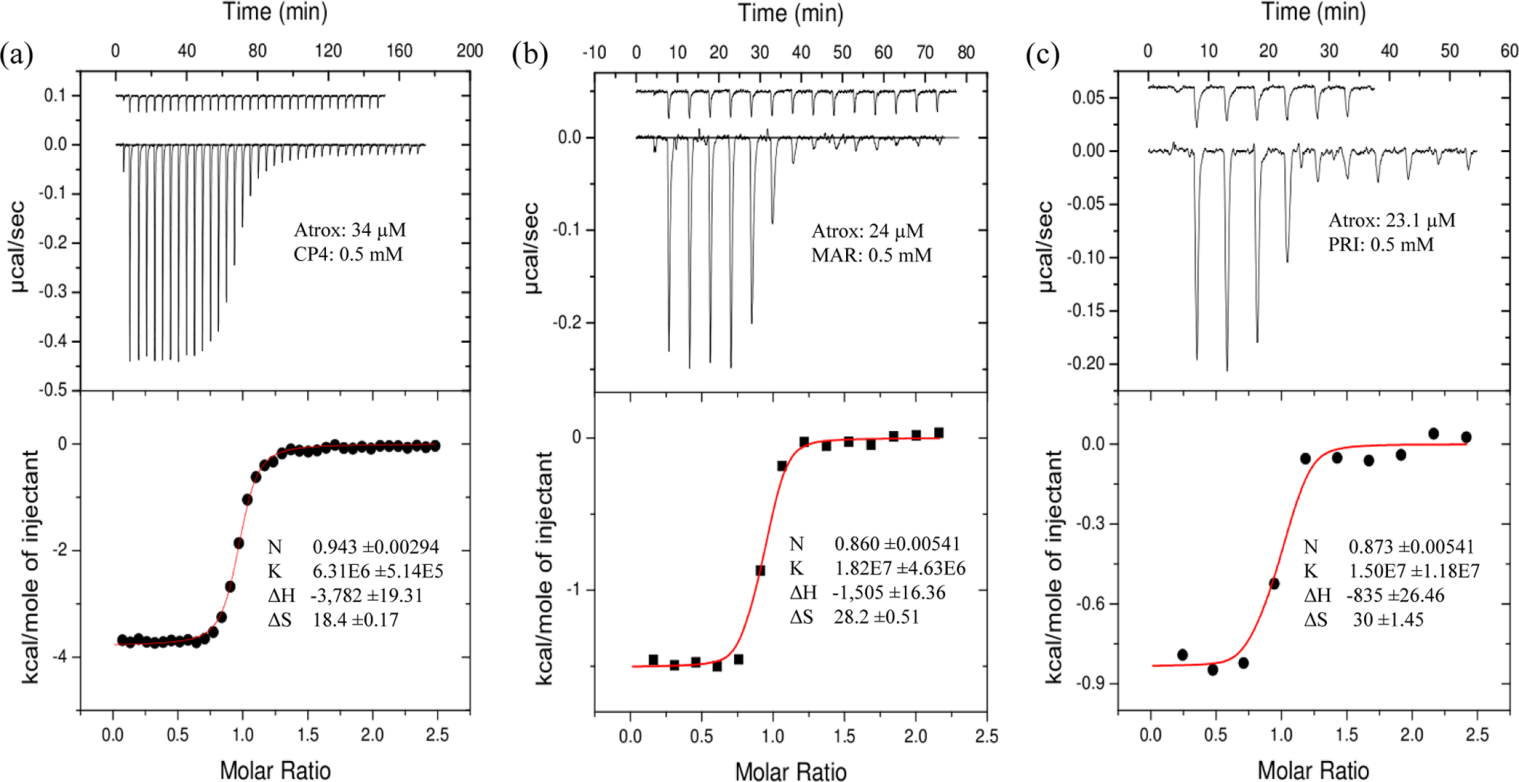
ITC curves at 25 °C and the computed thermodynamics parameters for Atr-I and (a) CP4, (b) MAR, and (c) PRI. *N* is the stoichiometry of the binding, *K* is the association constant (*i.e.*, the inverse of the dissociation constant, *K_i_*), Δ*H* is the enthalpy variation, and Δ*S* is the entropy variation. Toxin and inhibitor concentrations are also included.

To characterize the SVMP inhibitors, we selected six compounds bearing a hydroxamate group, previously described as broad-spectrum metalloprotease inhibitors (Figure 2), based primarily on their potency against ADAMs and MMPs.^21^ Then we evaluated them through kinetics assays for Atr-I and Leuc-a, selected as models of SVMPs (Figure 3), and through ITC for Atr-I and three of these selected inhibitors (Figure 4).

The compounds tested exhibited high potency against the toxins, with IC_50_ values ranging from of 20 nM to 12 μM, which is consistent with literature values for other metalloproteases.^126−139^ The IC_50_ values of the inhibitors against Atr-I were mostly similar, ranging from 20 to 160 nM, except for COL, which had a lower potency with an IC_50_ value of 8 μM. Similar trends were observed for Leuc-a, with IC_50_ values ranging from 20 to 690 nM. An exception was observed for BAT, which had lower potency against Leuc-a than Atr-I, with an IC_50_ value of 12 μM, presenting a greater difference (240 times) between the two toxins.

BAT, MAR, and PRI have been extensively studied for their inhibitory potency against medically important metalloproteases,^129,131−133,140−145^ as they have reached clinical studies. However, there are considerable variations in IC_50_ values between experiments. In the case of BAT, up to 100-fold differences in IC_50_ values were observed, even in assays performed with the same protein, probability due to the specific conditions of each assay (eq 1). Although *K*_*i*_ values tend to show less variation, there is limited information about them. It is worth noting that BAT, MAR, and PRI have previously been tested against effects induced predominantly by SVMPs in whole venoms from viperid species known to contain high levels of these enzymes.^146−148^ When these findings are considered alongside the sequence and structural similarities between class PI SVMPs and those of classes PII and PIII, they suggest that these compounds likely exhibit inhibitory activity across all SVMP classes.

On the other hand, there are only a few reported IC_50_ values for COL, MMP, and CP4 against MMPs. Overall, the reported IC_50_ and *K*_*i*_ values for the six compounds evaluated here are within 10-fold of the ones reported for other metalloproteases, in agreement with their broad-spectrum behavior.^32,126,127,129,131,132,136,137,140,141,149−155^ Occasionally, these values extend to a 100-fold range, which is reasonable given the inherent variability in IC_50_ values under different experimental conditions. Furthermore, despite similarities in the active site, considerable variability exists among the protein targets, possibly contributing to these observed variations.

To assess the correlation between our experimental results and the various MM/PBSA-based scorings, the experimental Δ*G* values were estimated from the IC_50_ values (Table 1) with the eq 1. We considered the *K*_m_ constant to be much larger than the substrate concentration used in the kinetics assay, as it was not possible to determine its precise values due to the limited aqueous solubility of the substrate. The resulting Δ*G* values based on the IC_50_ data range between ∼−7 and ∼−11 kcal/mol (Table 1).

**Table 1:** IC_50_, *K*_*i*_, and Δ*G* Values of Each Inhibitor to Atr-I and Leuc-a^a^

	Atr-I				Leuc-a			
inhibitors	ITC *K*_*i*_ (μM)	ITC Δ*G*	IC_50_ (μM)	ΔG^*^ (kcal/mol)	Hill coef.	IC_50_ (μM)	ΔG^*^ (kcal/mol)	Hill coef.
BAT	-	-	0.05 ± 0.03	–10.00	0.85	12.00 ± 1.00	–6.75	4.91
COL	-	-	8.00 ± 2.00	–7.00	2.28	6.00 ± 2.00	–7.16	1.65
CP4	0.16 ± 0.01	–9.54	0.02 ± 0.01	–10.56	2.37	0.21 ± 0.11	–9.16	0.55
MAR	0.06 ± 0.01	–12.46	0.08 ± 0.01	–9.74	2.05	0.69 ± 0.02	–8.45	0.78
MMP	-	-	0.05 ± 0.01	–10.02	1.79	0.36 ± 0.17	–8.84	1.34
PRI	0.07 ± 0.05	–13.00	0.16 ± 0.01	–9.32	0.77	0.02 ± 0.01	–10.56	1.96

a *K*_*i*_ and Δ*G* values were obtained from ITC, and IC_50_ with confidence intervals, Δ*G*, and Hill coefficients obtained by kinetic assays for the toxins/inhibitors. ΔG* values are estimated of IC_50_ values with data obtained by kinetic assays.

After obtaining the kinetic results and confirming the compounds’ inhibitory activities, we performed ITC assays aiming to obtain more accurate affinities measures and other thermodynamic parameters. Due to the limited availability of purified toxins and the low solubility of the inhibitors, the results were obtained only with Atr-I and CP4, MAR, and PRI (Table 1 and Figure 4). These compounds were prioritized since they have already reached phase III clinical trials (MAR and PRI)^21,24^ and present higher solubility and potency against the toxins in the kinetics assays, increasing the likelihood of obtaining high-quality data. Despite also being potent and having reached phase III of clinical studies,^156^ BAT was not included due to limited solubility.

The Δ*G* values (in kcal/mol) for the inhibitors (Table 1 and Figure 4), indicate that PRI (−13.0) exhibits the highest potency against Atr-I, followed by MAR (−12.46) and CP4 (−9.54). The higher accuracy of the CP4 results, compared to MAR and PRI, is evident in the one-order-of-magnitude difference in the standard deviation of the association constant (*K*) measurements. This improvement is due to the greater number of measurement points obtained before reaching enzyme saturation, when the inhibitor addition produces a similar heat amount when added to only the buffer or the saturated protein solution, thus resulting in a more accurate binding model fitting of the binding isotherm (Figure 4). Ideally, employing a proper quantity of toxin to gather curves with point numbers similar to those achieved for CP4, along with conducting repeated experiments, would yield more robust statistical analyses and, consequently, more reliable data for the other compounds. However, we were unable to conduct additional ITC experiments due to the limited quantities of purified toxins.

The differences between the Δ*G* obtained through the kinetics assays and ITC results (Table 1) could be attributed to the experimental limitations listed above, particularly the inability to determine the *K*_m_ value of the enzymatic substrate, considering the approximation *K*_*i*_ ≈ IC_50_. Nevertheless, both assays confirmed that these compounds are potent inhibitors against these enzymes.

One of the main advantages of ITC is to obtain a direct measure of the enthalpic and the entropic contributions of the Δ*G* of binding, allowing to determine the thermodynamics of binding processes and providing valuable information for further optimization of the inhibitors (Figure 4).^157^ CP4 exhibited a larger enthalpic contribution (−3.8 kcal/mol) than PRI (−0.8 kcal/mol), despite their structural similarities (Figure 2, Table S1). MAR’s enthalpic contribution was approximately 40% (−1.5 kcal/mol) of that obtained for CP4. However, CP4 had a lower entropic contribution (18.4 cal/K/mol) than PRI and MAR (30 and 28.2 cal/K/mol, respectively). MAR, with its more flexible structure, was expected to have a smaller entropic contribution than PRI and CP4, although its value was close to that of PRI. These entropic contributions were much larger than the enthalpic contributions for the binding free energy of these inhibitors, resulting in a larger binding Δ*G* value for PRI, despite its much lower enthalpic variation compared to MAR and CP4. We also calculated the entropic contribution using computational methods. However, the limited number of experimental data did not allow for well-grounded correlation calculations with experimental entropic terms. It is also important to consider that direct comparison with theoretical calculations poses other significant challenges, as experimental data encompass essential factors not considered in theoretical assessments, such as entropy variations related to active site desolvation. Moreover, changes in enthalpy and entropy often exhibit weak correlations with experimental data on binding free energies, leading some investigators to suggest that designing ligands based solely on free energy considerations might be more effective than attempting separate optimizations of enthalpy or entropy for specific ligands.^158^

Another important contribution of the ITC experiments is the determination of stoichiometry to be 1 for Atr-I/ligand complexes, supporting our molecular simulation models. This result also agrees with the crystallographic structures of MAR with other metalloproteases.^23,91^

### Structural Analysis of the MD Simulations

After confirming the compound’s inhibitory activities *in vitro*, docking and MD simulations of toxins and the six inhibitor complexes were performed to propose binding modes and compute their binding free energies.

A significant fraction of the most favorable AutoDock docked structures exhibit (His)_3_Zn···OCNHO(hydroxamate)···HOOC-Glu_143_ interactions and place one hydrophobic inhibitor group within the *S*_1_′ pocket of the SVMP enzymes. Both characteristics are conserved among hydroxamate inhibitors bound to metalloproteases as previously found in crystallographic studies and computational QM/MM studies.^159−162^ However, we highlight that the use of the tuned atomic charges (Figure S1) was necessary to observe frequent ZBG···Zn contacts. Even after applying of the refined charge scheme, we still observed docking poses that lack any ZBG···Zn interactions and/or show an empty S1′ pocket. Furthermore, AutoDock scoring functions give similar values for the various poses regardless of their SVMP-ligand contacts.

Therefore, to improve the accuracy of the docking calculations, we rescored the best AutoDock poses with an SQM/MM protocol (described in Methods). Then, we visually inspected the ten top-scored poses for each complex to select the most likely binding modes, favoring poses containing the hydroxamate/zinc ion interactions and one nonpolar group placed in the S1′ pocket. Except for CP4 and PRI, we identified more than one binding mode among the best-scored poses, and each of these binding modes was selected as initial structures for the subsequent MD simulations. We also evaluated the inhibitors CP4 and PRI in their *zwitterionic* form (Figure S4), which is a possible charge configuration of these compounds in aqueous solution. Thus, 13 different configurations for each protein, varying in the type of inhibitor or the binding mode, were selected for carrying out MD simulations (Figure S5).

Despite all the selected inhibitors bearing a hydroxamate group as ZBG, which demanded a considerable effort for MM parametrization, they present two different scaffolds and diverse groups in the variable regions of the molecules, even among those with the same scaffold, as reflected by low Tanimoto pairwise coefficients in general (Table S1). Thus, this compound set can provide valuable information about specific toxins/inhibitors interactions and is a suitable set for testing the performance of the MM-PBSA-based scoring functions.

As mentioned in Methods, to ensure that the Zn coordination sphere and the inhibitor’s binding mode remain stable along the MD simulations, we derived a set of MM parameters including explicit Zn/inhibitor bonds, with the hydroxamate group acting as a bidentate Zn ligand. These explicit bonds keep the hydroxamate slightly asymmetrically anchored to the metal center (equilibrium Zn^2+^•••O distances of 2.08 and 2.13 Å) and H-bonded to the protonated Glu_143_. These MM parameters succeeded in maintaining the zinc environment (Table 2), the Zn···ZBG contacts, and the ligands’ binding modes (Tables S2 and S3) quite stable during all the simulations, an extremely important feature for proper computational scoring. Concerning the overall structure of the complexes, the MD simulations revealed that it has moderate fluctuations. Besides that, the systems in most of the simulations were very stable, with an RMSD average of approximately 0.9 Å (Figures S6 and S7), as expected for these toxins, mainly due to their relatively small size, compact shape, and presence of three disulfide bonds.^27^

**Table 2:** Mean RMSD Values (in Angstroms) of all MD Simulations for Each Toxin for the Selected Regions, or the Whole Protein^a^

toxins	Atr-I	Leuc-a
region/position	RMSD (Å)	RMSD (Å)
total	0.90 ± 0.14	0.90 ± 0.13
100–108	0.64 ± 0.23	0.55 ± 0.16
148–179 (Ω-loop)	0.89 ± 0.20	0.76 ± 0.17
156–164	0.87 ± 0.28	0.60 ± 0.16
154–162	1.01 ± 0.30	0.69 ± 0.20
zinc environment	0.35 ± 0.12	0.37 ± 0.11

a The zinc environment is defined by the amino acid residues His_142_, Glu_143_, His_146_, His_152_, the zinc ion, and the inhibitor molecule.

In addition to the overall analysis of protein stability in the simulations, we performed more focused mobility analyses on areas critical for protein–protein interactions between SVMPs and extracellular matrix proteins,^27^ as well as interactions with drug-like inhibitors (Tables 3 and 4). In our previous MD study with Atr-I/Leuc-a enzymes, we observed differences in the mobility of the Ω-loop. More specifically, we found that the Atr-I toxin exhibited greater mobility than Leuc-a. Interestingly, the same result is observed in the present MD trajectories of the SVMP/inhibitor complexes, the Ω-loop RMSD descriptors of Atr-I being in general above those of Leuc-a (Table 2). In addition, we noted disparities in the backbone folding and surface charge in the 100–108 loop of these two toxins.^28^ Other MD simulations showed flexibility differences in the Ω-loop at residues 156–165 and 167–175, located close to the active site, what may be linked to differences in the hemorrhagic activity of SVMPs.^33^ Moreover, marked structural differences in the Ω-loop have been reported among metalloproteinases with varying hemorrhagic activities.^24^ In a more recent and interesting study, a recombinant hemorrhagic toxin of Bap1, in which the Ω-loop 154–162 residues are replaced with those from the same region of the nonhemorrhagic toxin Leuc-a,^163^ preserves its proteolytic activity while its hemorrhagic activity is removed, highlighting the significance of this region for hemorrhagic activity. We analyzed the RMSD of this specific region (154–162) and found again greater mobility in the Atr-I toxin (1.01 ± 0.30) compared to Leuc-a (0.69 ± 0.20) (*p* < .001). These findings further support the hypothesis of a close relationship between the difference in mobility in this region and hemorrhagic activity.

**Table 3:** Main H-Bonds Toxins/Inhibitors Interactions^a^

	Atr-I		Leuc-a	
residue	interactions numbers/interacting complexes	mean interaction frequency (%)	interactions numbers/interacting complexes	mean interaction frequency (%)
Pro/Glu-106	13/10	61	12/8	59
Thr107	3/3	49	3/3	41
Ile108	12/12	92	13/13	80
Gly109	23/13	140	20/13	128
Ala111	-	-	3/3	22
Glu143	24/13	127	20/13	127
Pro/Asp-168	7/7	41	10/7	57
Leu170	10/10	74	9/7	86

a Interaction numbers indicate the total of interactions detected with a given residue, considering all complexes, and may include more than one interaction in same complex, while the interacting complexes indicate in how many of the complexes a given interaction was observed. Residues with more than 100% of mean interaction frequency presented interactions with more than one hydrogen donor or acceptor of the inhibitor molecule, in at least one MD simulation. Only residues with a number of interacting complexes greater than two are listed.

**Table 4:** Main van der Waals Toxins/Inhibitors Interactions^a^

	Atr-I			Leuc-a		
residue	interactions numbers/interacting complexes	mean interaction frequency (%)	mean interaction energy	interactions numbers/interacting complexes	mean interaction frequency (%)	mean interaction energy
Pro/Glu-106	13/10	107	–1.58	-	-	-
Ile108	20/13	127	–1.74	19/13	125	–1.84
Leu/Ile-110	8/7	80	–0.96	8/7	68	–0.94
Met/Arg-135	4/4	46	–0.73	-	-	-
Ile/Val-138	8/8	93	–1.09	9/9	87	–1.07
His142	16/12	132	–3.04	17/13	125	–3.20
His152	7/5	39	–1.01	13/9	62	–1.20
Val/Thr-169	8/7	77	–1.09	-	-	-
Leu170	19/12	113	–2.06	19/13	113	–2.12
Pro/Leu-174	4/4	78	–0.97	-	-	-
Lys/Phe-176	-	-	-	4/4	77	–1.09

a Residues with more than 100% of mean interaction frequency presented interactions with more than one inhibitor nonpolar fragment of the inhibitor molecule in at least one MD simulation *of a specific* toxin/inhibitor *complex*. Only residues with a number of interacting complexes greater than two are listed.

**Figure 5:**
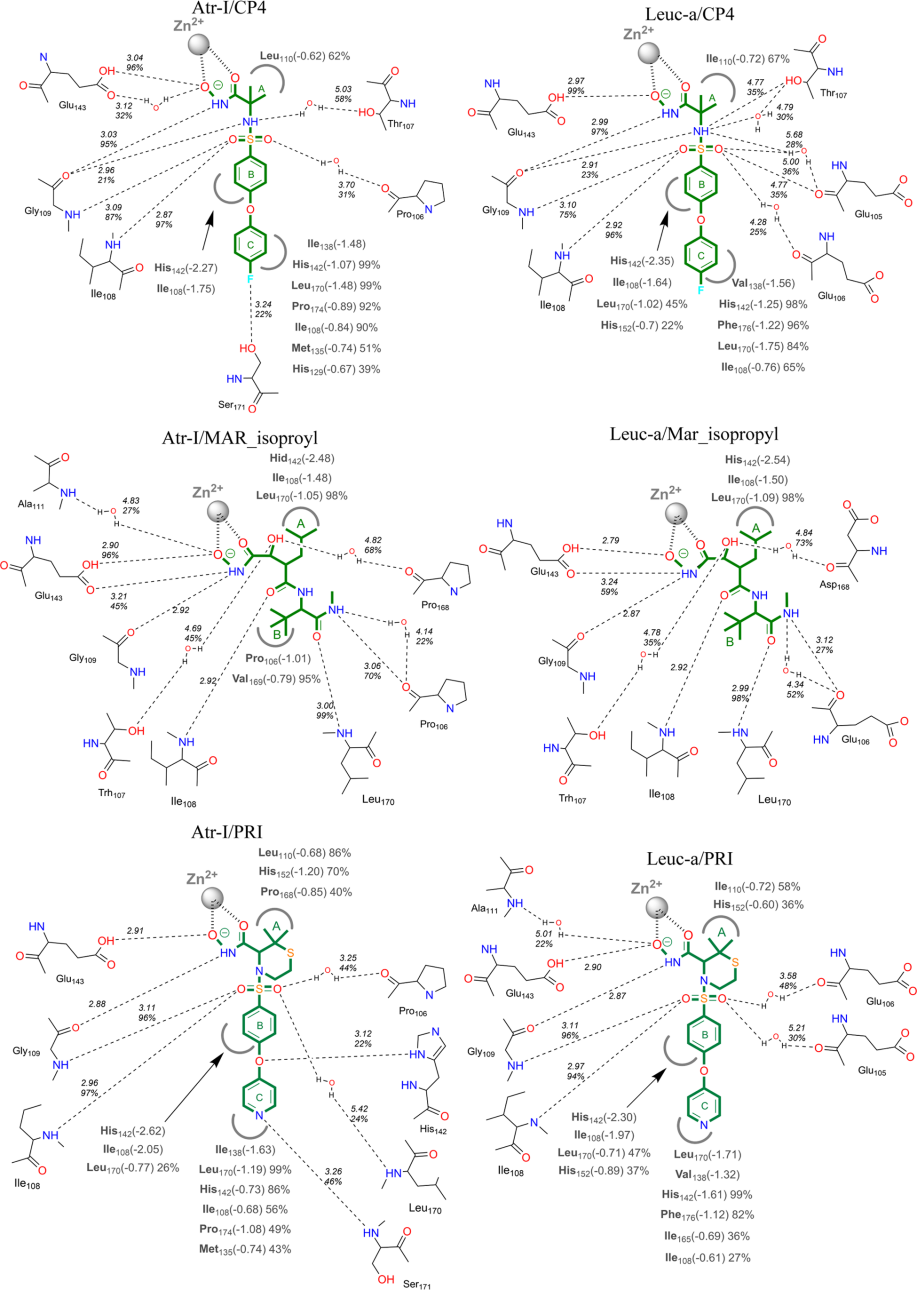
Schematic 2D depiction of main inhibitor/toxin interactions observed in MD simulations of Atr-I and Leuc-a with the inhibitors employed in ITC assays. Hydrogen bonds are denoted by dotted lines, with average interatomic distance values (in Å) and the percentage of interaction duration provided. van der Waals interactions are illustrated by semicircles, featuring residue names and corresponding values for the percentage of interaction duration and interaction energy in kcal/mol. Nonpolar groups of the inhibitors are identified by letters. Interaction percentage values are omitted for interactions that occurred throughout the entire MD simulation.

**Figure 6:**
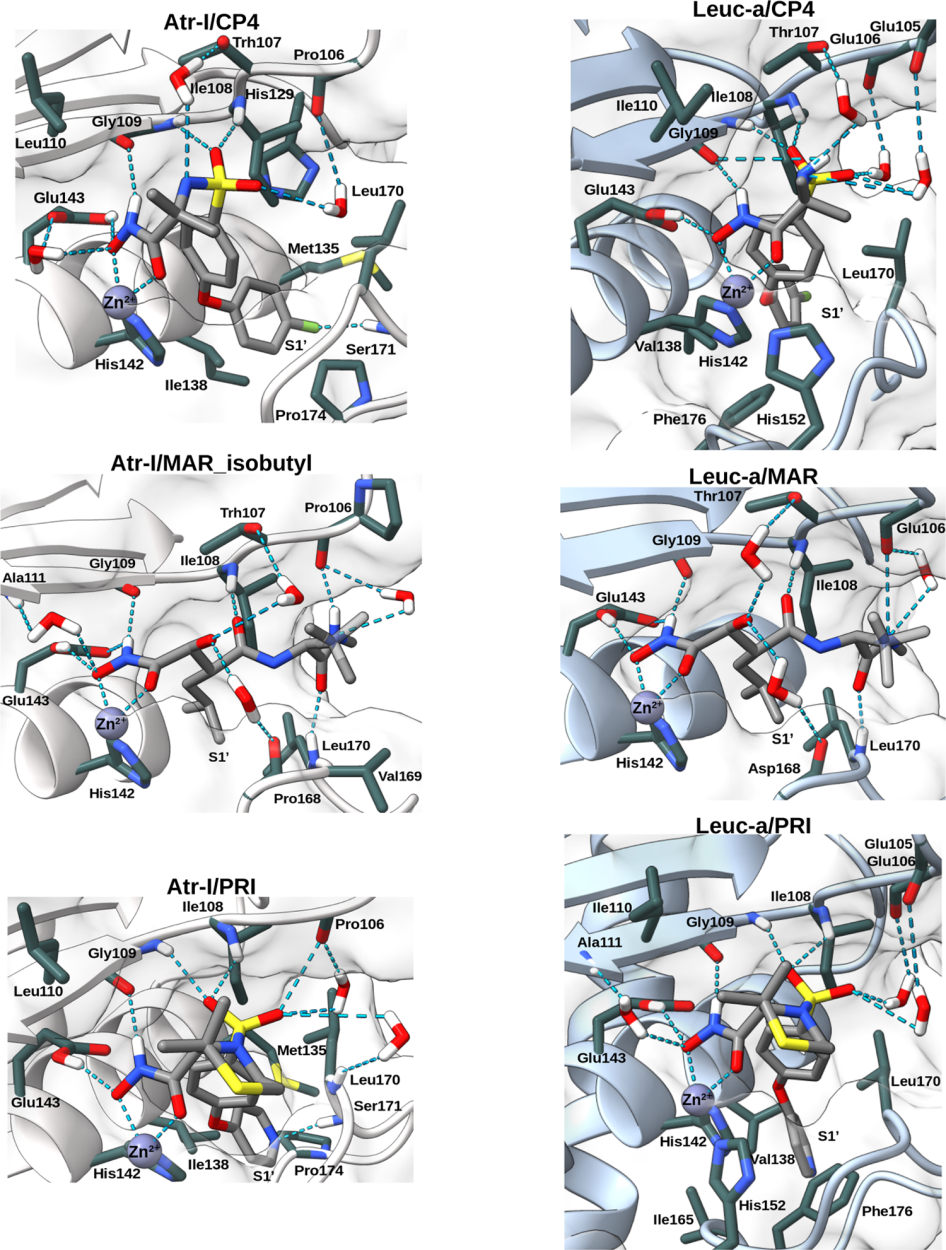
Interacting amino acid residues of Atr-I (white cartoon) or Leuc-a (blue cartoon) with inhibitors. Protein carbon atoms involved in hydrogen-bonding and van der Waals interactions are depicted in dark green, and carbon atoms from the inhibitors in dark gray. Backbone atoms are shown in stick representation only when necessary to illustrate hydrogen bonding. Hydrogen atoms participating in hydrogen bonds are selectively displayed. The dotted lines in cyan represent hydrogen bonds shown. The figures only show interactions that occur for more than 20% of the duration of the MD simulations. The structures of the toxin/inhibitor complexes were obtained by clustering with the RMSD values of the inhibitors and amino acids involved in the interactions. Figures displaying hydrogen bonds involving water molecules were produced by superimposing these structures with structures from other poses generated in the simulations, chosen based on illustrative criteria, and showing only the water molecules involved in these interactions.

To better understand the stability and interaction patterns observed in the MD simulations of our complexes, we identified the most relevant enzyme/ligand interactions observed in ≥20% of the MD length and classified them into polar (H-bonds) and nonpolar ones. The corresponding two-dimensional (2D) representations are illustrated in Figure 5 (featuring inhibitors from the ITC assays) and Figure S8 (incorporating all inhibitors used in the enzyme kinetics assays in all binding modes evaluated), and the three-dimensional (3D) representations in Figures 6 and S9. We also determined the “interaction number” of some SVMP residues, besides the number of complexes with interactions, by counting the occurrences of amino acid residues interacting with hydrogen donor or acceptor atoms of the inhibitor, as well as nonpolar regions of the inhibitor, across all the simulations. For instance, if an amino acid residue has a polar interaction number of 13, it corresponds to an average of one H-bond interaction per simulation. Overall, we observed similar patterns of interactions in most of the Leuc-a and Atr-I simulations, with comparable lifetimes calculated as a percentage of the total MD simulations and interaction numbers (Tables 3 and 4). These similarities occurred even at the S1′ pocket, which plays a crucial role in substrate specificity in metzincins.^20,164,165^ Initially, one could expect differences in substrate interactions between these two toxins within this pocket, considering their partially distinct substrate specificities and the associated variance in hemorrhagic activity. However, these results align with our previous work, which demonstrated similar shapes and sizes of this pocket in both toxins during MD simulations.^28^ Furthermore, these results underscore that the variation in hemorrhagic activity between the two toxins does not stem from differences in the catalytic site, but rather from another region of the toxin, such as the Ω-loop.

Most of the H-bond interactions in the complexes occur with the main chain atoms of the residues Pro_106_(Atr-I)/Glu(Leuc-a)_106_ (O), Ile_108_ (N), Gly_109_ (O and N), Pro/Asp_168_ (O), and Leu_170_(N) and the side chain oxygens of Glu_143_, which is essential for catalytic activity (Figures 5, 6, S8 and S9, and Table 3). In addition, Thr_107_ establishes hydrogen bonds mediated by water molecules in three complexes for each toxin, and the residue Thr_169_ in one complex with Leuc-a, without water molecules. These data agree with other studies showing that the toxins-peptides or toxin-peptidomimetics interactions occur in a conformation similar to β-sheet structures,^165^ mainly mediated by main-chain atoms, and with the crystallographic structures of BAT and MAR complexed with metalloproteases.^22,91,165^ The predominance of these ligand interactions with main-chain atoms is important for the broad-spectrum activity of these inhibitors. Hydrophobic interactions occur mainly in the S1′ pocket through the residues His_142_, Leu_170_, Ile_108_, Ile_138_(Atr-I)/Val_138_(Leuc-a) for bulkier nonpolar groups (Figures 5, 6, S8 and S9, and Table 4). Outside this pocket, hydrophobic interactions occur mainly with the residues Ile/Leu_110_, His_146_, His_152_, Ile_108_, and Leu_170_, for Leuc-a. For Atr-I, besides these residues, the nonpolar residues Pro_106_ and Val_169_ were frequently involved in polar and nonpolar interactions. In Leuc-a, these positions are occupied by Glu_106_ and Thr_169_, which present negatively charged and polar side chains respectively, preventing them from participating in nonpolar interactions. Additionally, we observed a difference in the hydrophobic interactions in position 152, which only occurred in the Atr-I complexes, even though this position is occupied by a His residue in both toxins.

In summary, the observed pattern of enzyme–inhibitor contacts in both toxins explains the broad-spectrum activities of the inhibitors against ADAMs, SVMPs, and MMPs, as the active site amino acids variations present a limited impact in the formation of H-bonds, which occur with the main-chain atoms and with side-chain oxygens with the catalytic Glu residue, found in all metzicins clan members. Similarly, the sequence variations have also a minimal effect in the main hydrophobic interactions within the highly hydrophobic S1’ pocket, despite the variations in size and shape among enzymes of this clan.

### Computational Scoring of the SVMP Inhibitors

As described in Methods, the scoring functions of the enzyme–inhibitor affinities are composed of interaction and distortion energy contributions as well as entropic terms. To evaluate the interaction energy, we employed MM-PB(GB)BSA and QM/MM-PB(GB)SA methodologies, using two nonbonding parameter sets (hfe and iod) for the MM calculations. For the QM/MM scorings, three SQM methods (DFTB, DFTB3, and PM6) were used to describe the Zn coordination environment including only the hydroxamic ZBG or the whole inhibitor molecule in the QM region. The same methods were used to measure the influence of the enzyme and inhibitors’ distortion energy while the entropic contributions were computed with four different approximations. This resulted in a total of 216 different scoring function combinations, which were assessed by means of correlation tests with the experimental data retrieved from the kinetic assays. For the SVMP/inhibitor complexes that were simulated in various states (*i*.*e*., by varying the inhibitor group within the S1’ pocket and/or the charge configuration of the inhibitor), the comparison with experimental data is performed by selecting the most favorable scoring among the various states.

**Figure 7:**
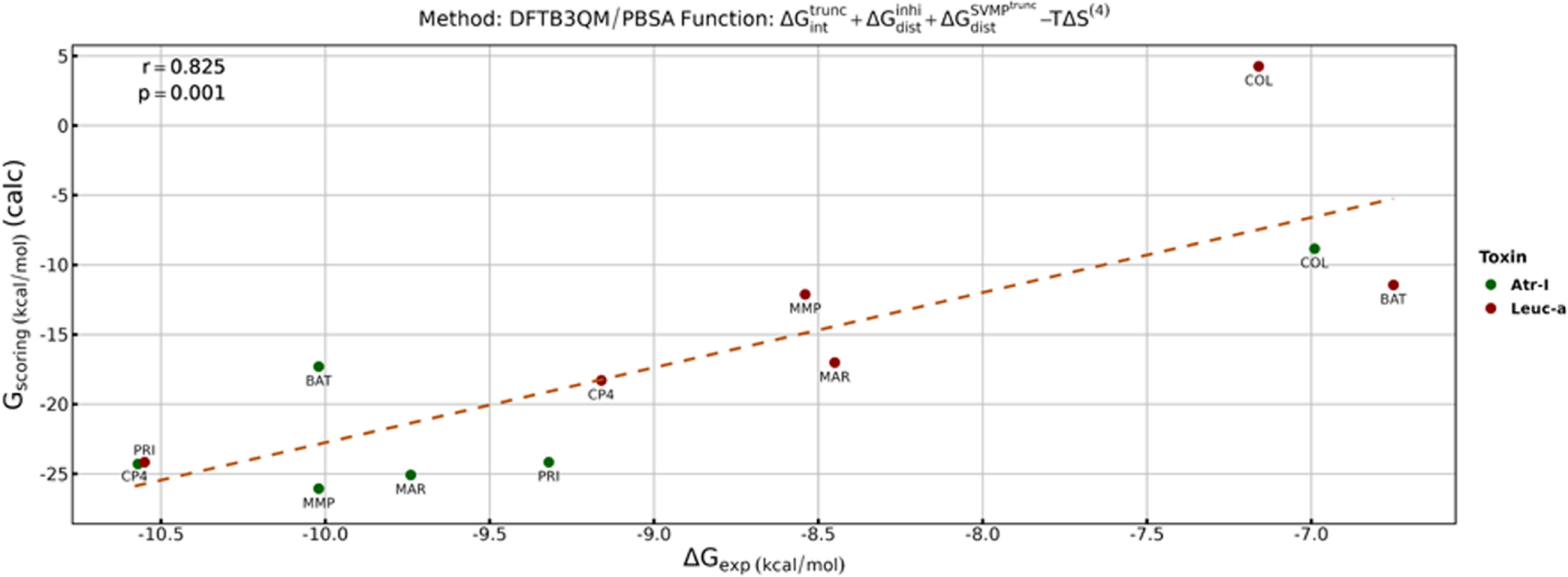
Graphics with the best correlation plots between computational calculations and kinetics assays for the two toxins.

Table 5 shows the top ten scoring function combinations that present the best correlations with the kinetics assays, while in Table S4 we applied a cutoff *p*-value of 0.01. Figure 7 displays a comparison between experimental data and the values calculated with the scoring functions that produce the best results for the two toxins.

**Table 5:** Top Ten Tested Scoring Function Combinations and *r* and *p*-Values of Correlation Tests with Kinetics Assay Data for Both Toxins^a^

EΔ*G*/solv. methods	scoring function	*r*-value	*p-*value
DFTB3/PBSA	Δ*G*_int_ + Δ*G*_dis_^inhi^ + Δ*G*_dis_^SVMP_trunc^ – *T*(Δ*S*^(4)^)	0.83	0.001
PM6/PBSA	Δ*G*_int_ – *T*(Δ*S*^(2)^)	0.82	0.001
PM6/PBSA	Δ*G*_int_^trunc^ – *T*(Δ*S*^(2)^)	0.82	0.001
DFTB/PBSA	Δ*G*_int_^trunc^ – *T*(Δ*S*^(2)^)	0.81	0.001
PM6/PBSA	Δ*G*_int_^trunc^ + Δ*G*_dis_^inhi^ – *T*(Δ*S*^(2)^)	0.80	0.002
PM6/PBSA	Δ*G*_int_^trunc^ + Δ*G*_dis_^inhi^ + Δ*G*_dis_^SVMP_trunc^ – *T*(S^(Δ4)^)	0.79	0.002
PM6/PBSA	Δ*G*_int_	0.79	0.002
DFTB(QM_small_)/GBSA	Δ*G*_int_^trunc^ + Δ*G*_dis_^inhi^ + Δ*G*_dis_^SVMP_trunc^ – *T*(S^(Δ4)^)	0.79	0.003
MM/GBSA_iod	Δ*G*_int_^trunc^ + Δ*G*_dis_^inhi^ + Δ*G*_dis_^SVMP_trunc^ – *T*(S^(Δ4)^)	0.78	0.003
PM6/PBSA	Δ*G*_int_ + Δ*G*_dis_^inhi^ – *T*(Δ*S*^(2)^)	0.78	0.003

a The “small” suffix means that only the hydroxamate group of the inhibitors was included in the QM region.

Most functions that yielded better correlations for both toxins (with *p*-values lower than 0.01) combined the interaction energy with the entropic terms including RRHO and conformational contributions, Δ*S*^(2)^ = Δ*S*_RRHO_ + Δ*S*_conform_^inhi^ and Δ*S*^(4)^ = Δ*S*_RRHO_ + Δ*S*_conform_^inhi^ + Δ*S*_conform_^SVMP^. Although some of the scoring functions produced good results with just these terms, most of them also included the distortion energies of the inhibitor and the truncated enzyme. The best energy methods corresponded to SQM methods, that is, DFTB/PB(GB)SA, DFTB3/PBSA, and PM6/PBSA. Interestingly, the SQM scorings are more reliable when the “small” QM region is used, which provides a proper description of the Zn···ZBG interaction while the rest of the SVMP···inhibitor contacts are described by the MM force field in a balanced manner.

In addition to assessing the functions with the highest correlation values for both toxins, we conducted a more comprehensive evaluation of the impact of various energetic terms on the scoring functions. This involved calculating the averages of the *r* values obtained from correlation analyses for each function across the different energy methods analyzed. Subsequently, we compared these averages for each function against all others using Student’s *t*-test (Tables S5 and S6). Therefore, *p*-values smaller than 0.05 indicate a significant statistical difference.

These analyses revealed again that the Δ*G*_int_ alone is frequently insufficient to obtain a good correlation coefficient, especially for Leuc-a, regardless of whether the full or truncated protein structure is used. We comparatively evaluated the use of truncated forms of the toxins because the latter could allow a decrease in the computational cost of calculating the free energy of binding and reduce the uncertainty of calculating the protein’s distortion energy and the entropy change during the binding process.

For both Atr-I and Leuc-a systems, the combination of distortion energies and Δ*G*_int_—particularly the enzyme’s truncated form for Atr-I and the inhibitor’s distortion energy for Leuc-a—along with the entropic terms Δ*S*^(2)^ and Δ*S*^(4)^, significantly improved the correlation with experimental data (Tables 5 and S4). Remarkably, the presence of multiple free energy terms in almost all of the top-performing scoring functions, assessed for both toxins and through the averaging of correlation coefficients, suggests that, despite the observed similarities in hydrogen bonding, van der Waals interactions, and interactions involving the zinc atom and the hydroxamate group of the inhibitors across all simulations, the relative affinity between inhibitors and enzymes is significantly influenced by the distortion energies of both the inhibitors and toxins, as well as by the entropy variations occurring during the binding process (Tables 5 and S4).

We also evaluated the impact of different methods on the correlation performance of functions using the same approach as the comparison of function terms explained above (Tables S7 and S8). Notably, for both Atr-I and Leuc-a, the PM6/PBSA method yielded the best results in these analyses. Another noteworthy finding was the good performance of DFTB with both solvation methods when only the small region of the inhibitor was included in the QM area, producing significantly better outcomes than when the entire inhibitor was incorporated into the QM region.

While our study identified these patterns in the scoring functions and their correlation with experimental data for the inhibitors analyzed against two specific enzymes, further investigation is required to determine whether these patterns extend to other medically relevant enzymes. Studies involving additional enzymes and inhibitors would yield more robust data on the broader applicability of these scoring functions, enhancing their reliability in drug discovery efforts.

Besides focusing on the relative affinity of the SVMP inhibitors, the scoring functions can also determine the most favorable binding mode and/or inhibitor configuration. To this end, we adopted a consensus approach merging the information retrieved from the best scorings according to the affinity rankings. Thus, Table 6 presents the preferences of the scoring functions for the different binding modes of the toxin/inhibitor complexes, which are based on different nonpolar groups of inhibitors within the S1’ pocket. More specifically, we determined the most favorable binding mode for each inhibitor/toxin complex, highlighted in bold in Table 6, by analyzing the scores from the scoring functions that obtained a *p*-value less than or equal to 0.1 in the correlation tests and calculating the frequency at which each function yielded a favorable score for a given binding mode, compared to other binding modes within a specific toxin/inhibitor complex. This evaluation was performed across all binding modes of enzyme–inhibitor complexes formed by the two enzymes and the six inhibitors. The frequencies were then converted into percentages. Therefore, for each enzyme–inhibitor complex, the total preferences for different binding modes sum to 100%. In general, we noticed that the preferred binding mode was consistent for both toxins, except for BAT, which may be related to the larger difference in affinity between the two toxins. Despite the similarity in the shape and volume of the S1’ pocket,^28^ in this case, the Atr-I toxin favored the phenyl group in the S1’ pocket, while Leuc-a favored the isobutyl group. Also noteworthy are the Atr-I complexes with COL and MMP, which showed a 100% preference for the isobutyl and phenyl groups, respectively. However, obtaining crystallographic complex structures would be necessary to confirm these findings.

**Table 6:**
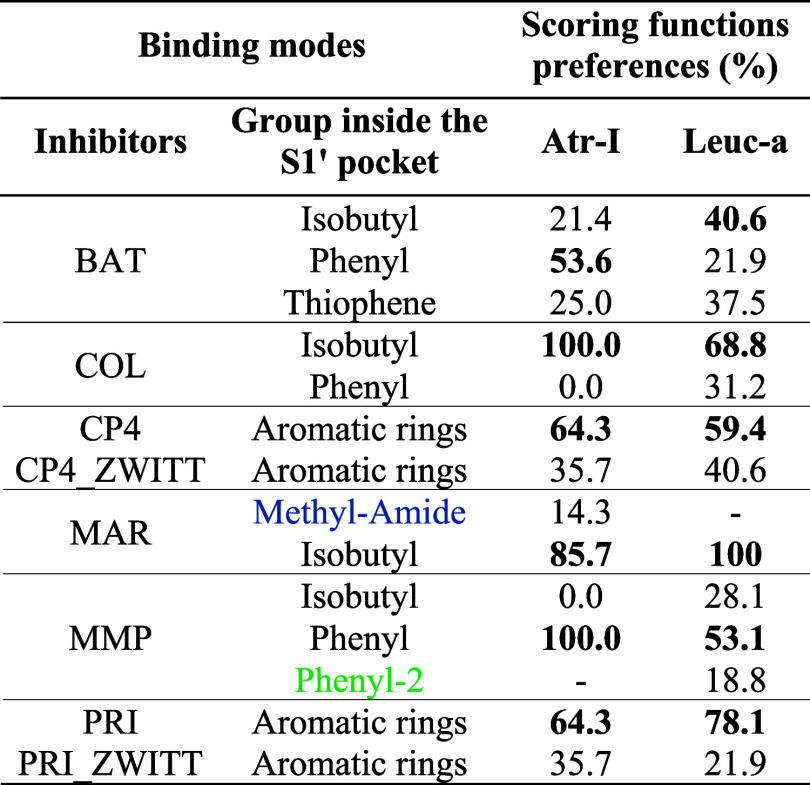
Preferential Binding Modes of Toxins/inhibitors Complexes^a^

a Inhibitors binding modes used in the MD simulations with both toxins (black), or with only Atr-I (blue), and Leuc-a (green), with the percentage of the most favorable ones, based on the scoring functions results, highlighted in bold. ZWITT stands for zwitterionic configuration.

## Conclusions

In this study, we identified and described six drug-like broad-spectrum metalloprotease inhibitors for the SVMPs Atr-I and Leuc-a, with IC_50_ values ranging from 20 to 690 nM, demonstrating their potential efficacy in the micromolar or nanomolar range. Through ITC experiments, we successfully determined the binding affinity of Atr-I with inhibitors CP4, MAR, and PRI, marking the first report of ITC results with nonrecombinant metalloproteases. Notably, MAR and PRI, which have already undergone human testing via oral administration, exhibit promising potential for auxiliary serum therapy in accidents with venomous snakes.

Employing MD simulations, we elucidated the main interactions between the inhibitors and SVMPs, unraveling the molecular details behind the broad-spectrum behavior of metalloprotease hydroxamate inhibitors. On the other hand, our investigation did not reveal a distinct pattern of difference in binding affinities or toxin/inhibitor interactions between the two toxins that could be linked to their varying bleeding activity.

Furthermore, our evaluation of MM-PBSA-based scoring functions underscored the significance of including distortion energies and entropic terms, affirming the superior accuracy of QM/MM over MM methods in computing the binding affinity of metalloprotease/inhibitor complexes provided that a minimal QM region comprising the Zn coordination shell is selected. More particularly, combining the DFTB method with the GBSA solvation method yields satisfactory results. Moreover, we identified the possibility of utilizing truncated forms of the protein, offering reliable results while reducing computational costs. Our methodological approach not only allows for comparisons that incorporate other corrective terms, which can be computed using the same or alternative methodologies, but also has the potential to refine virtual screening results.

## Data Availability

Inputs and outputs of docking and molecular dynamics simulations, inhibitors/toxins interactions, free energy and entropic calculations are freely available at: https://zenodo.org/records/13687928.

## Supporting Information Available

The file contains the details of the atoms cluster used in docking simulations, the derived parameters of the zinc environment used in the MD simulations, additional analyses of the molecular simulations, and the comparison between the scoring functions’ performance (PDF)

## Abbreviations

SVMP: snake venom metalloproteinases
ADAM: a disintegrin and metalloproteinases
MMP: matrix metalloproteinases
ZBG: zinc-binding group
BAT: batimastat
MAR: marimastat
PRI: prinomastat
CP4: CP471474
MM/PBSA: molecular mechanics Poisson–Boltzmann surface Area
MM/GBSA: molecular mechanics generalized Born surface area
TI: thermodynamic integration
FEP: perturbation free energy
PB: Poisson–Boltzmann equations
GB: generalized Born
MD: molecular dynamics
QM: quantum mechanical
SCC-DFTB: self-consistent charge density functional tight-binding
SQM: semiempirical quantum mechanical
PM6: parametric method 6
Atr-I: atroxlysin-I
Leuc-a: leucurolysin-a
FRET: fluorescence resonance energy transfer
COL: collagenase-I inhibitor
MMP: mmp-III inhibitor
ITC: isothermal titration calorimetry
DMSO: dimethyl sulfoxide
PDB: protein data bank
vdW: van der Waals
B3LYP: Becke 3-parameter Lee–Yang–Parr
GAFF: Generalized Amber Force Field
PCM: polarizable continuum model
RMSD: root-mean squared deviations
TNCG: truncated-Newton conjugate gradient
DOF: degrees of freedom
RRHO: rigid rotor and harmonic oscillator
PDF: probability density function
RMSF: root-mean squared flexibility
IOD: ion-oxygen distance
HFE: hydration free energy
