# Supplementary material for: Assessing the Interactions between Snake Venom Metalloproteinases and Hydroxamate Inhibitors Using Kinetic and ITC Assays, Molecular Dynamics Simulations and MM/PBSA-Based Scoring Functions
Source: ACS Omega. 2024 Dec 10;9(51):50599–621. doi: 10.1021/acsomega.4c08439 (PMC11684173; doi:10.1021/acsomega.4c08439)
Supplement: Supplementary file 1 — ao4c08439_si_001.pdf [file ao4c08439_si_001.pdf]

SUPPLEMENTARY MATERIAL

Assessing the Interactions between Snake Venom Metalloproteinases and Hydroxamate Inhibitors Using Kinetic and ITC Assays, Molecular Dynamics Simulations and MM/PBSA-based Scoring Functions

Raoni A. de Souza[a], Natalia Díaz[b], Luis G. Fuentes[c], Adriano Pimenta[d], Ronaldo A. P. Nagem[d], Carlos Chávez-Olórtegui[d],Francisco S. Schneider[e], Franck Molina[e], Eladio F. Sanchez[a], Dimas Suárez[b], Rafaela S. Ferreira[d*]*

[a] Rua Conde Pereira Carneiro 80, Dept. de Pesquisa e Desenvolvimento, Fundação Ezequiel Dias, Belo Horizonte, 3010-010, Minas Gerais, Brazil.

[b] Avda Julián Clavería 8, Dept. de Química Física y Analítica, Universidad de Oviedo, Oviedo, 33006, Asturias, Spain.

[c] Carretera Sacramento s/n, Dept. de Química y Física, Universidad de Almería, Almería, 04120, Andalucía, Spain.

[d] Avenida Antônio Carlos 6627, Dept. De Bioquímica e Imunologia, Universidade Federal de Minas Gerais, Belo Horizonte, 31270-901, Minas Gerais, Brazil.

[e] 1682, Rue de la Valsière, Sys2Diag, Cap Delta, Montpellier, 34184, Occitanie, France.

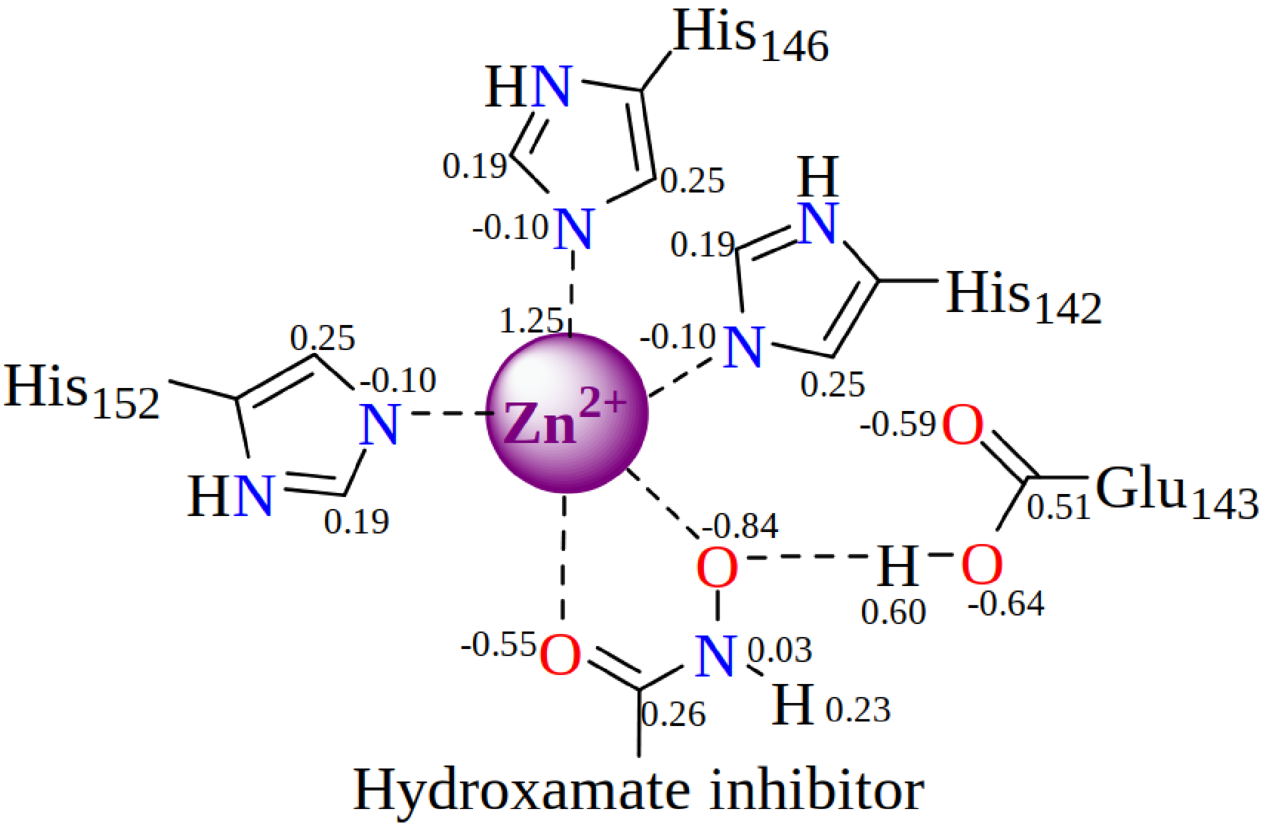

Figure S1. Atoms cluster used to compute the atomic charges of the Zn^{2+} and residues coordinated to this ion for docking simulations.

a)

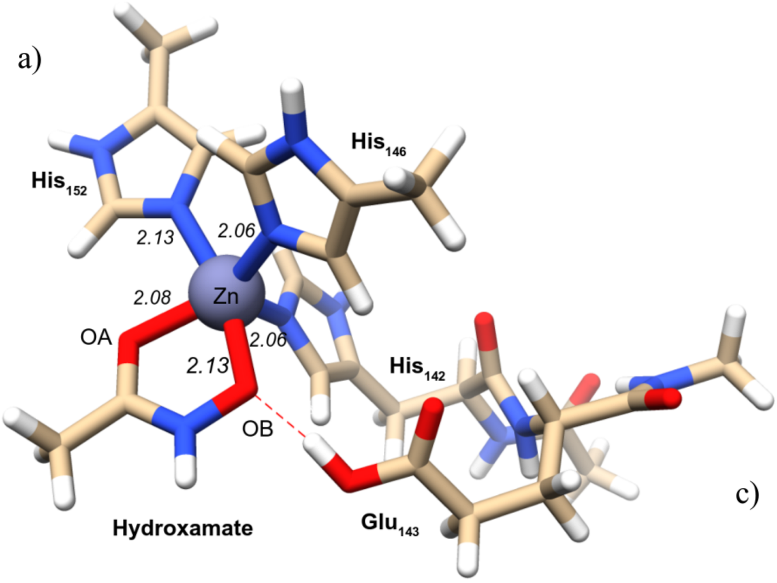

b)

Bond angles	
His146@Nε2···ZN···His152@Nε2	94º
His146@Nε2···ZN···His142@Nε2	119º
His142@Nε2···ZN···His152@Nε2	88º
His142@Nε2···ZN···OA	113º
His146@Nε2···ZN···OA	128º
His152@Nε2···ZN···OA	87º
His142@Nε2···ZN···OB	96º
His146@Nε2···ZN···OB	90º
His152@Nε2···ZN···OB	163º

c)

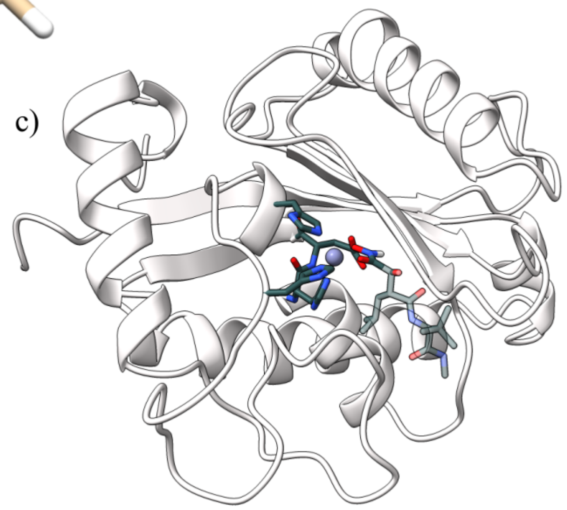

Figure S2. a) B3LYP/6-31G(d) PCM optimized structure of the Zn cluster model built from the PDB 4Q1L structure and the docking pose with BAT to obtain reference parameters (bond distances and bond angles) for the bonded MM representation of the Zn environment. The values of the reference parameters in Angstroms, or b) in degrees, are also indicated. c) Additionally, the entire Atr-I protein complexed with MAR is depicted with the MM region represented as a cartoon, and the QM region highlighted in a ball-and-stick representation. The inhibitor is displayed with an opaque region, representing the hydroxamate group used in the SCC-DFTB calculations (referred to as the small region), while the transparent portion indicates the additional structure included in all other QM calculations.

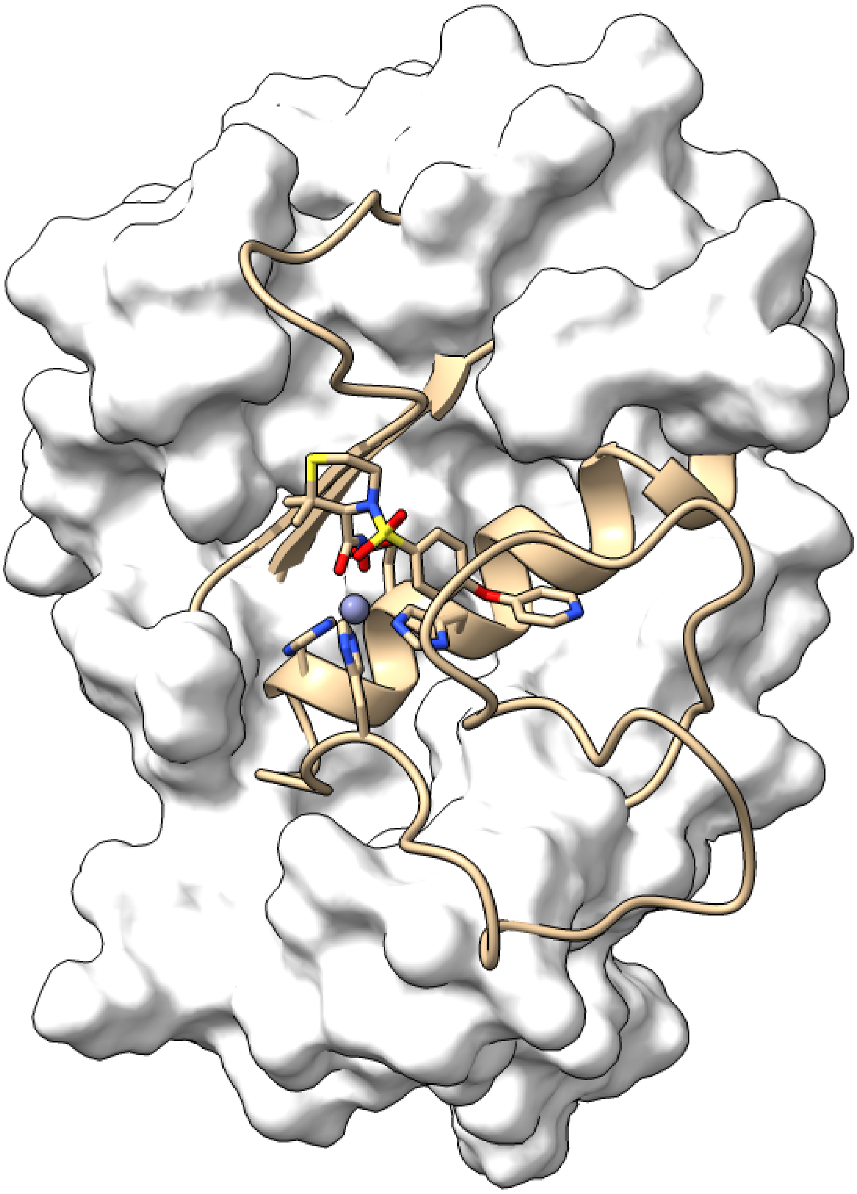

Figure S3. Atr-I/PRI complex structure highlighting, in ribbon representation, the truncated form used in the conformational entropy calculations and in some energy calculations. This same region was used for the calculations with Leuc-a.

Table S1. Tanimoto similarity coefficients between broad-spectrum metalloprotease inhibitors.

Compounds	BAT	COL	CP4	MAR	MMP	PRI
**BAT**	1.00	-	-	-	-	-
**COL**	0.56	1.00	-	-	-	-
**CP4**	0.49	0.57	1.00	-	-	-
**MAR**	0.68	0.64	0.55	1.00	-	-
**MMP**	0.77	0.68	0.54	0.85	1.00	-
**PRI**	0.53	0.53	0.75	0.49	0.50	1.00

PRI PRI ZWITT CP4 CP4 ZWITT

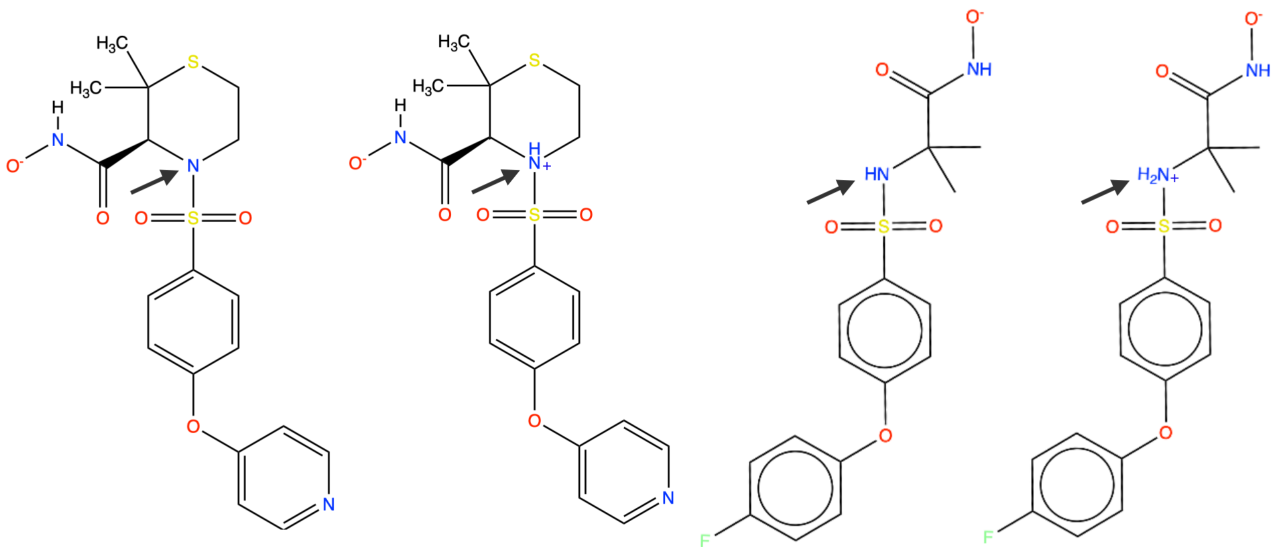

Figure S4. Two possible protonation states of the inhibitors PRI and CP4 used in the MD simulations. ZWITT stands for zwitterionic configuration. The arrows indicate the variation in charge distribution between the compounds.

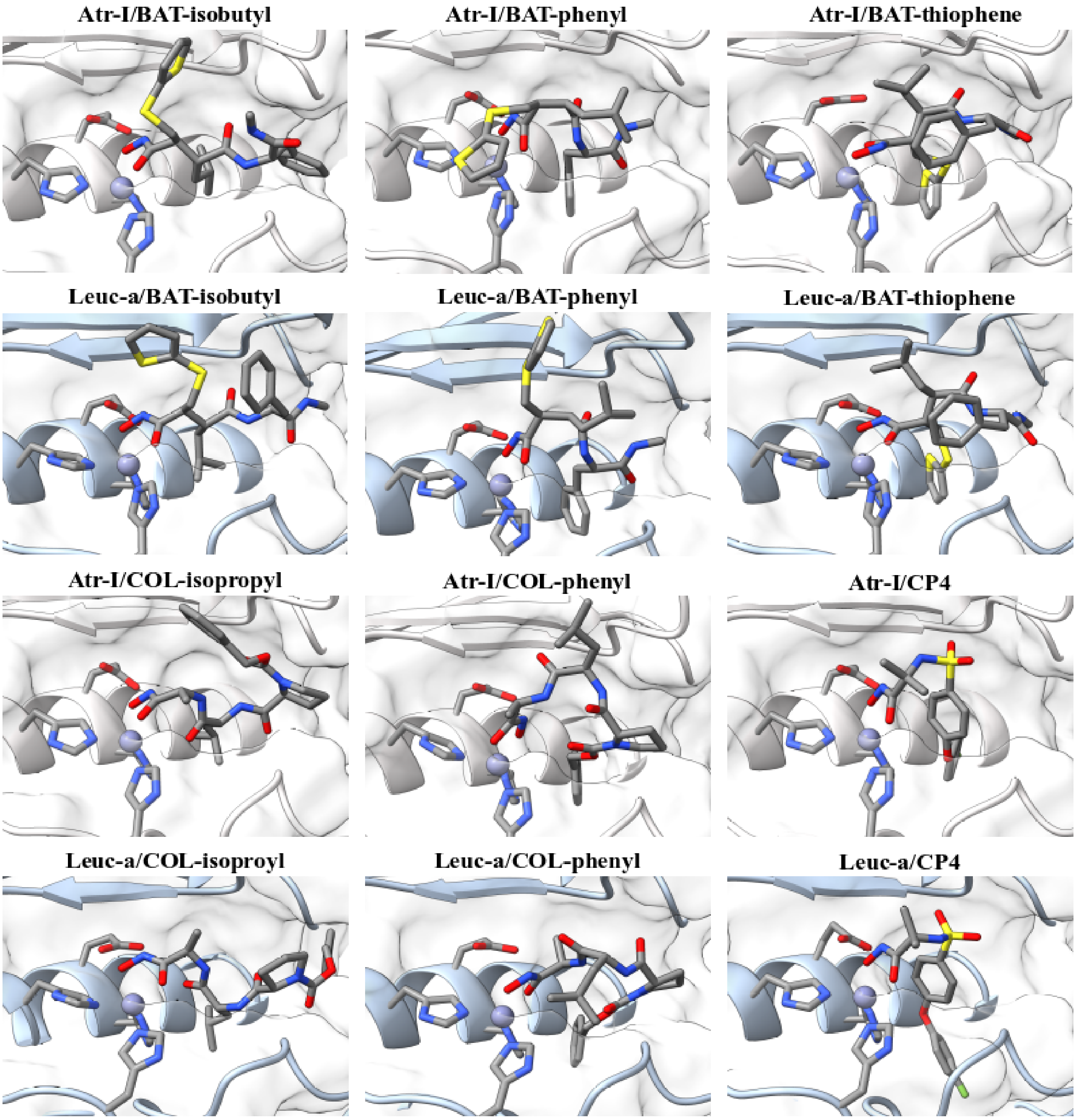

Figure S5. Initial structures of the toxin/inhibitor complexes obtained from docking simulations, used in MD simulations, with a focus on the active site. The molecular surface highlights the zinc atom (represented by a gray sphere) and the histidine residues coordinated with this atom, shown in stick representation, as well as the glutamic acid essential for catalytic activity. Inhibitors are also depicted in stick representation. The titles of the figures indicate the toxin, the inhibitor, and the chemical group of the inhibitor located in the S1' pocket. Part 1.

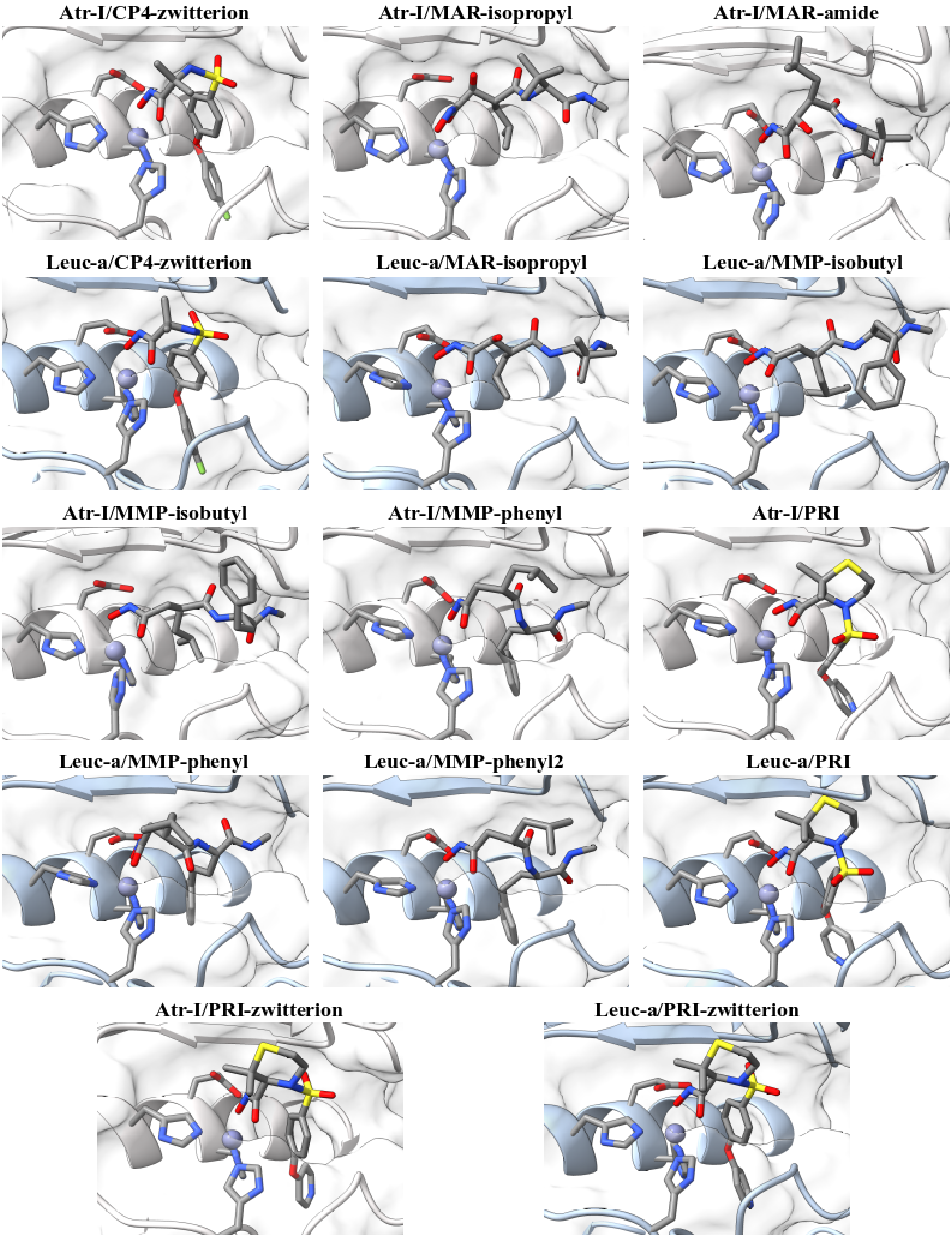

Figure S5. Part 2.

Table S2. Zn··ZBG contacts distance in MD simulations for Atr-I toxin.

**Interatomic contact**	**Zn^{+2}···O1**	**Zn^{+2}···O2**	**Zn^{+2}···His$_{142}$NƐ**	**Zn^{+2}···His$_{146}$NƐ**	**Zn^{+2}···His$_{152}$NƐ**	**Glu$_{143}$OƐ2···O1**
**Complex**			**Interatomic contact distance (Å)**			
BAT_isobutyl	2.15±0.05	2.08±0.05	2.04±0.05	2.05±0.06	2.16±0.06	3.22±0.29
BAT_phenyl	2.14±0.05	2.11±0.05	2.04±0.05	2.06±0.05	2.17±0.06	2.95±0.20
BAT_thiophene	2.15±0.05	2.07±0.05	2.05±0.05	2.05±0.06	2.16±0.06	3.25±0.30
COL_isopropyl	2.16±0.05	2.08±0.05	2.05±0.05	2.04±0.06	2.15±0.06	2.76±0.15
COL_phenyl	2.15±0.05	2.08±0.05	2.04±0.05	2.04±0.06	2.16±0.06	2.70±0.12
CP4	2.16±0.05	2.07± 0.05	2.04±0.05	2.06±0.06	2.16±0.06	3.06±0.22
CP4_zwitter	2.15±0.05	5.10±0.25	2.04±0.05	2.05±0.06	2.15±0.06	2.90±0.17
MAR_amide	2.15±0.05	2.08±0.05	2.04±0.05	2.06±0.06	2.16±0.06	2.88±0.17
MAR_isopropyl	2.15±0.05	2.08± 0.05	2.04±0.05	2.04±0.05	2.15±0.06	2.93±0.24
MMP_isobutyl	2.17±0.05	2.07± 0.05	2.05± 0.05	2.04± 0.05	2.15±0.06	3.37±0.26
MMP_phenyl	2.16±0.05	2.09±0.05	2.05±0.05	2.06±0.06	2.16±0.06	3.08±0.23
PRI	2.16±0.05	2.07±0.05	2.05±0.05	2.05±0.06	2.15±0.06	2.91±0.17
PRI_zwitter	2.15±0.05	5.68± 0.24	2.03± 0.05	2.06±0.06	2.15± 0.06	2.96±0.19

Atomic distance (mean value and standard deviation in Angstroms) in MD simulations between the oxygen atoms of the inhibitors' hydroxamate group and between Glu$_{143}$OƐ2 and the atom O1, which is linked to nitrogen of the hydroxamate group. The O2 atom is the other oxygen atom in the hydroxamate group.

Table S3. Zn···ZBG contacts distance in MD simulations for Leuc-a toxin.

**Interatomic contact**	**Zn^{+2}···O1**	**Zn^{+2}···O2**	**Zn^{+2}···His$_{142}$NƐ**	**Zn^{+2}···His$_{146}$NƐ**	**Zn^{+2}···His$_{152}$NƐ**	**Glu$_{143}$OƐ2···O1**
**Complex**			**Interatomic contact distance (Å)**			
BAT_isobutyl	2.15±0.05	2.07± 0.05	2.04±0.05	2.05± 0.06	2.15±0.06	2.81±0.13
BAT_thiophene	2.15±0.05	2.06± 0.05	2.03± 0.05	2.05± 0.06	2.15±0.06	2.85±0.15
BAT_phenyl	2.14±0.05	2.11± 0.05	2.04± 0.05	2.07± 0.05	2.16± 0.06	2.97±0.21
COL_isobutyl	2.15±0.05	2.08± 0.05	2.05± 0.05	2.05± 0.06	2.15± 0.06	2.83±0.14
COL_phenyl	2.15± 0.05	2.08± 0.05	2.04±0.05	2.05±0.06	2.15± 0.06	2.71±0.13
CP4	2.16±0.05	2.07± 0.05	2.05± 0.05	2.06±0.06	2.15±0.06	2.97±0.19
CP4_zwitter	2.16±0.05	5.53± 0.28	2.03±0.05	2.05±0.06	2.14±0.06	2.93±0.19
MAR	2.15± 0.05	2.08± 0.05	2.05±0.05	2.05±0.05	2.14±0.06	2.79±0.13
MMP_isobutyl	2.16±0.05	2.07± 0.05	2.06±0.05	2.05±0.05	2.14±0.06	2.88±0.15
MMP_phenyl	2.16±0.05	2.09± 0.05	2.05±0.05	2.06±0.06	2.16±0.06	3.07±0.24
MMP_phenyl2	2.15±0.05	2.09± 0.05	2.05±0.05	2.06±0.06	2.15±0.06	3.14±0.27
PRI	2.16±0.05	2.08± 0.05	2.05± 0.05	2.06±0.06	2.16±0.06	2.90±0.16
PRI_zwitter	2.15±0.05	5.72±0.34	2.04±0.05	2.06±0.06	2.14±0.06	2.95±0.19

Atomic distance (mean value and standard deviation in Angstroms) in MD simulations between the oxygen atoms of the inhibitors' hydroxamate group and between Glu$_{143}$OƐ2 and the atom O1, which is linked to nitrogen of the hydroxamate group. The O2 atom is the other oxygen atom in the hydroxamate group.

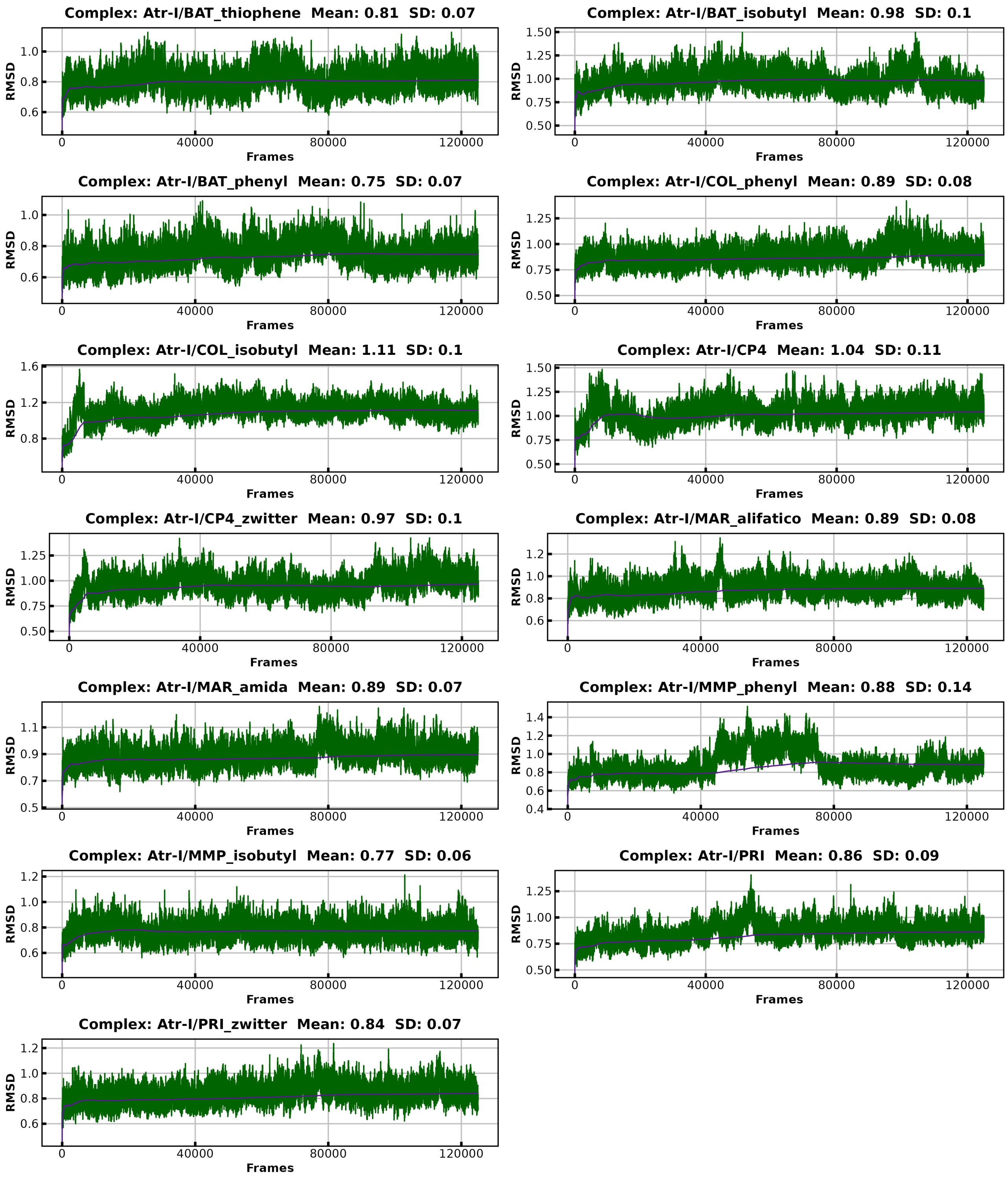

Figure S6. Time evolution of the RMSD computed for the backbone heavy atoms with respect to the first frame of the MD trajectories of Atr-I with the inhibitors. Average values and standard deviations (in Å) are included in the plot titles. The darkblue line indicates the cumulative mean.

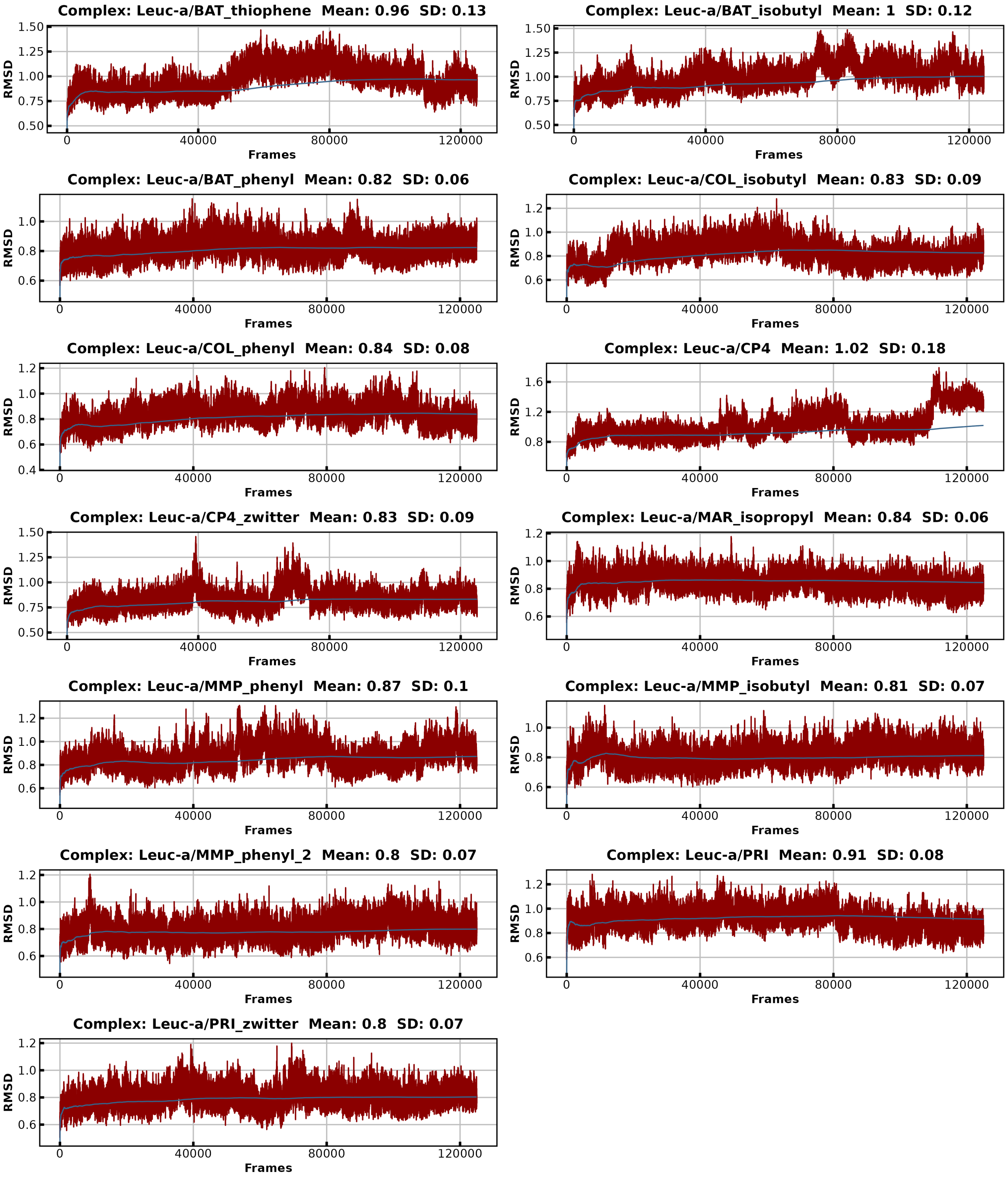

Figura S7. Time evolution of the RMSD computed for the backbone heavy atoms with respect to the first frame of the MD trajectories of Leuc-a with the inhibitors. Average values and standard deviations (in Å) are included in the plot titles. The dark blue line indicates the cumulative mean.

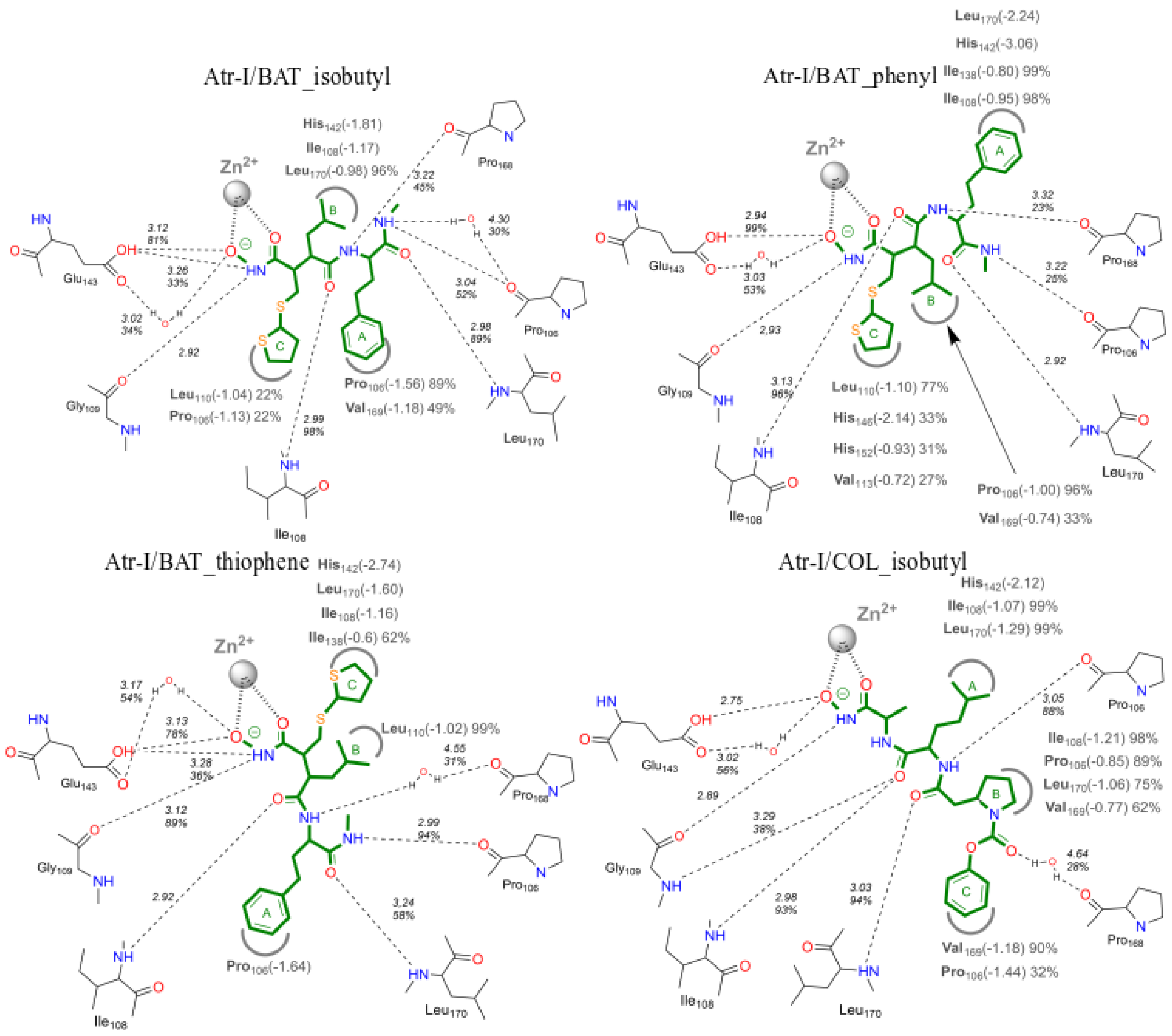

Figure S8. Schematic 2D representation of main inhibitor/toxin interactions observed in MD simulations of Atr-I and Leuc-a with the studied inhibitors. Hydrogen bonds are denoted by dotted lines, with average interatomic distance values (in Å) and the percentage of interaction duration provided. Van der Waals interactions are illustrated by semicircles, featuring residue names and corresponding values for the percentage of interaction duration and interaction energy in kcal/mol. Nonpolar groups of the inhibitors are identified by letters. Interaction percentage values are omitted for interactions that occurred throughout the entire MD simulation. Part 1.

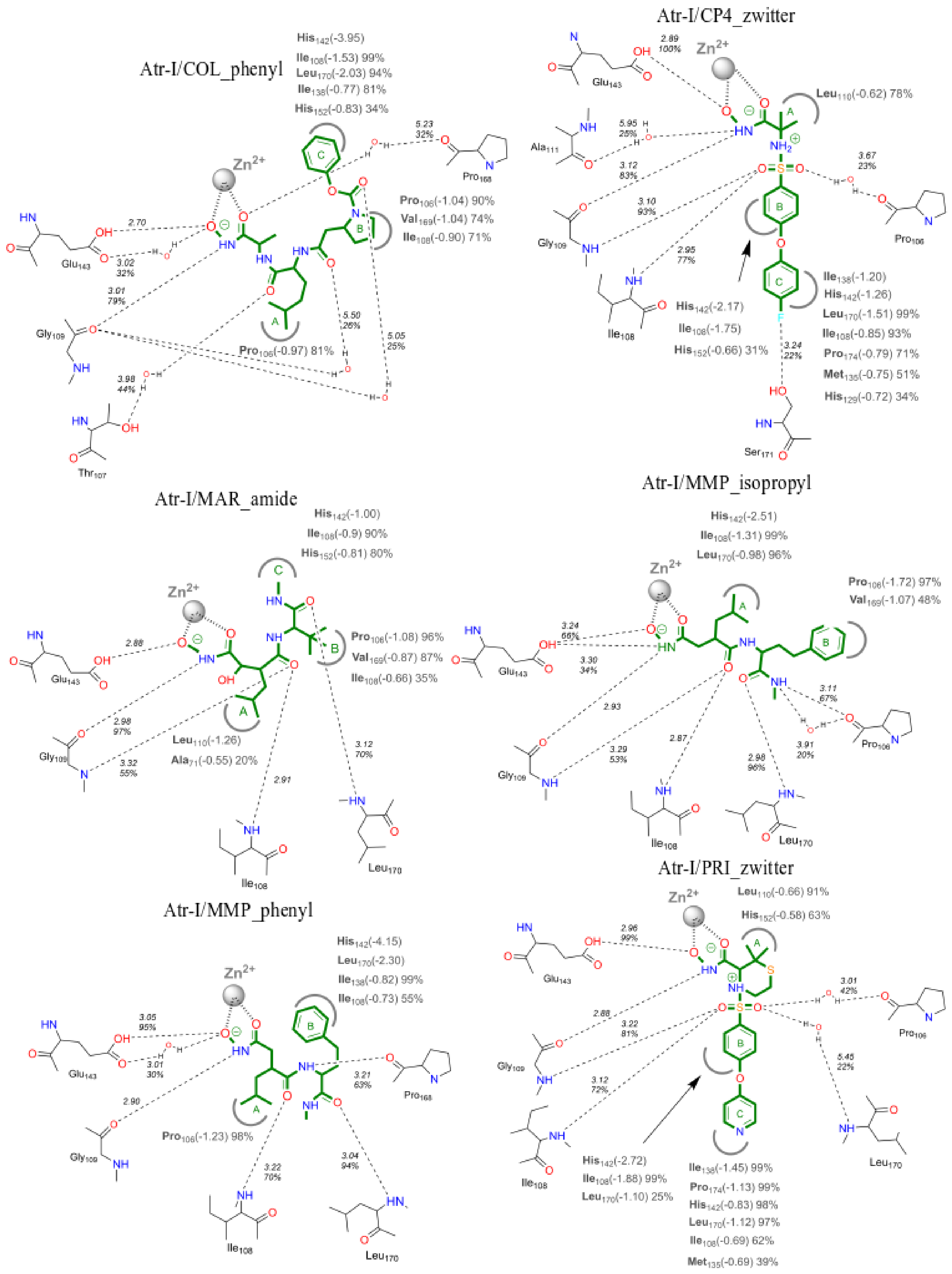

Figure S8. Part 2.

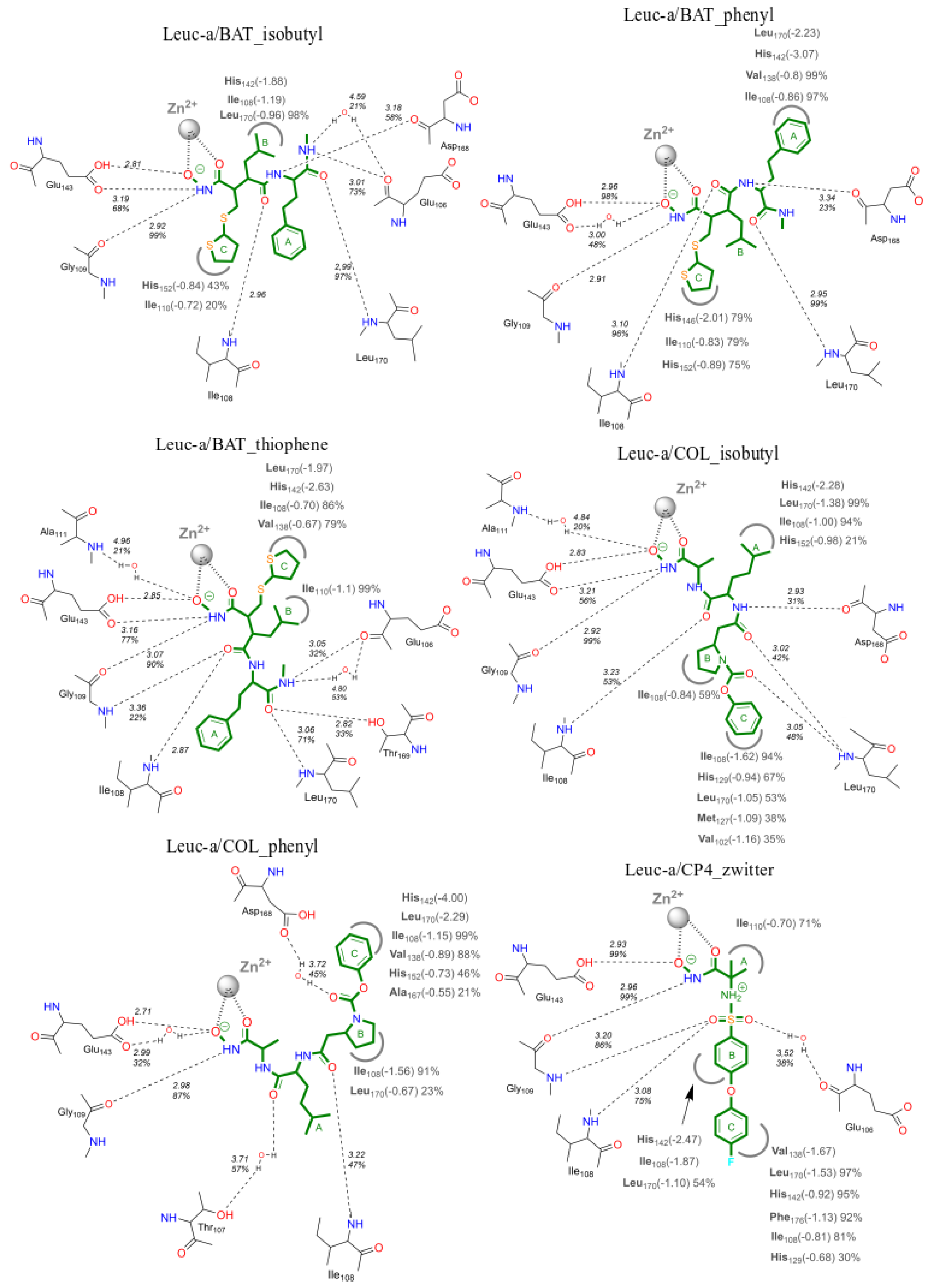

Figure S8. Part 3.

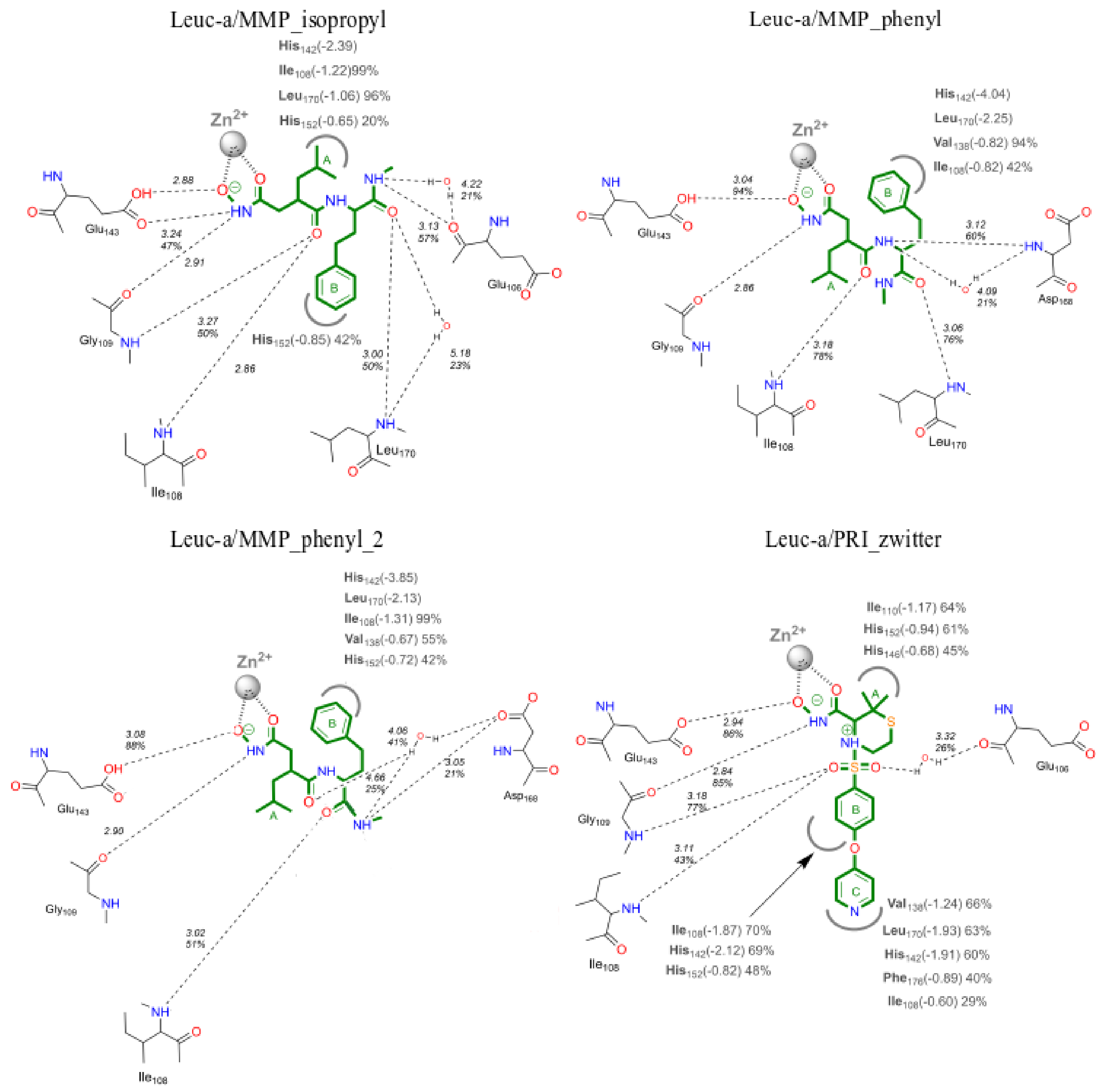

Figure S8. Part 4.

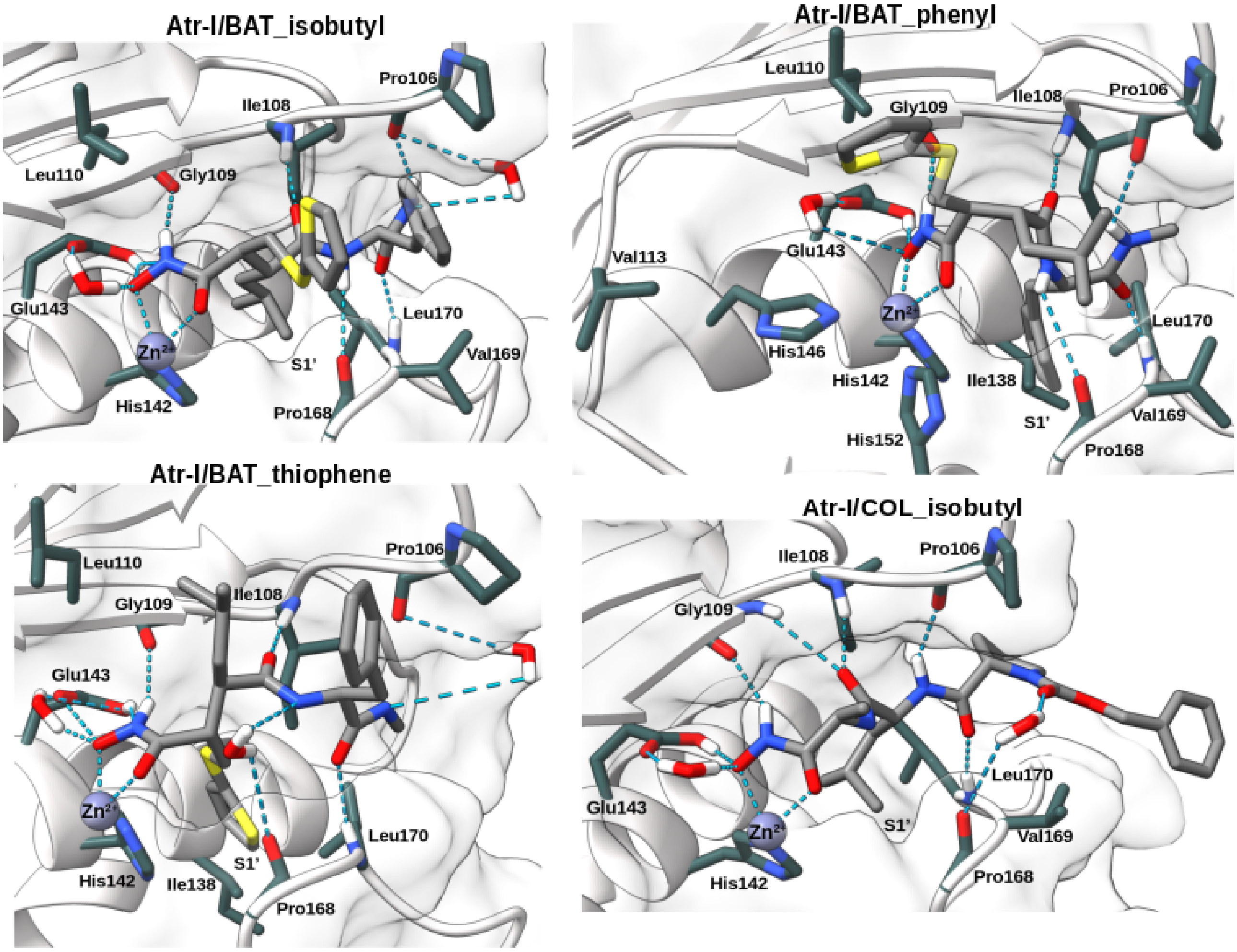

Figure S9. Interacting amino acid residues of Atr-I (white cartoon) or Leuc-a (blue cartoon) with inhibitors. Protein carbon atoms involved in hydrogen bonding and van der Waals interactions are depicted in dark green. Backbone atoms are shown in stick representation only when necessary to illustrate hydrogen bonding. Hydrogen atoms participating in hydrogen bonds are selectively displayed. The dotted lines in cyan represent hydrogen bonds. The figures only show interactions that occur for more than 20% of the duration of the MD simulations. Figures displaying hydrogen bonds involving water molecules were produced by superimposing these structures with structures from other poses generated in the simulations, chosen based on illustrative criteria, and showing only the water molecules involved in these interactions. Part 1.

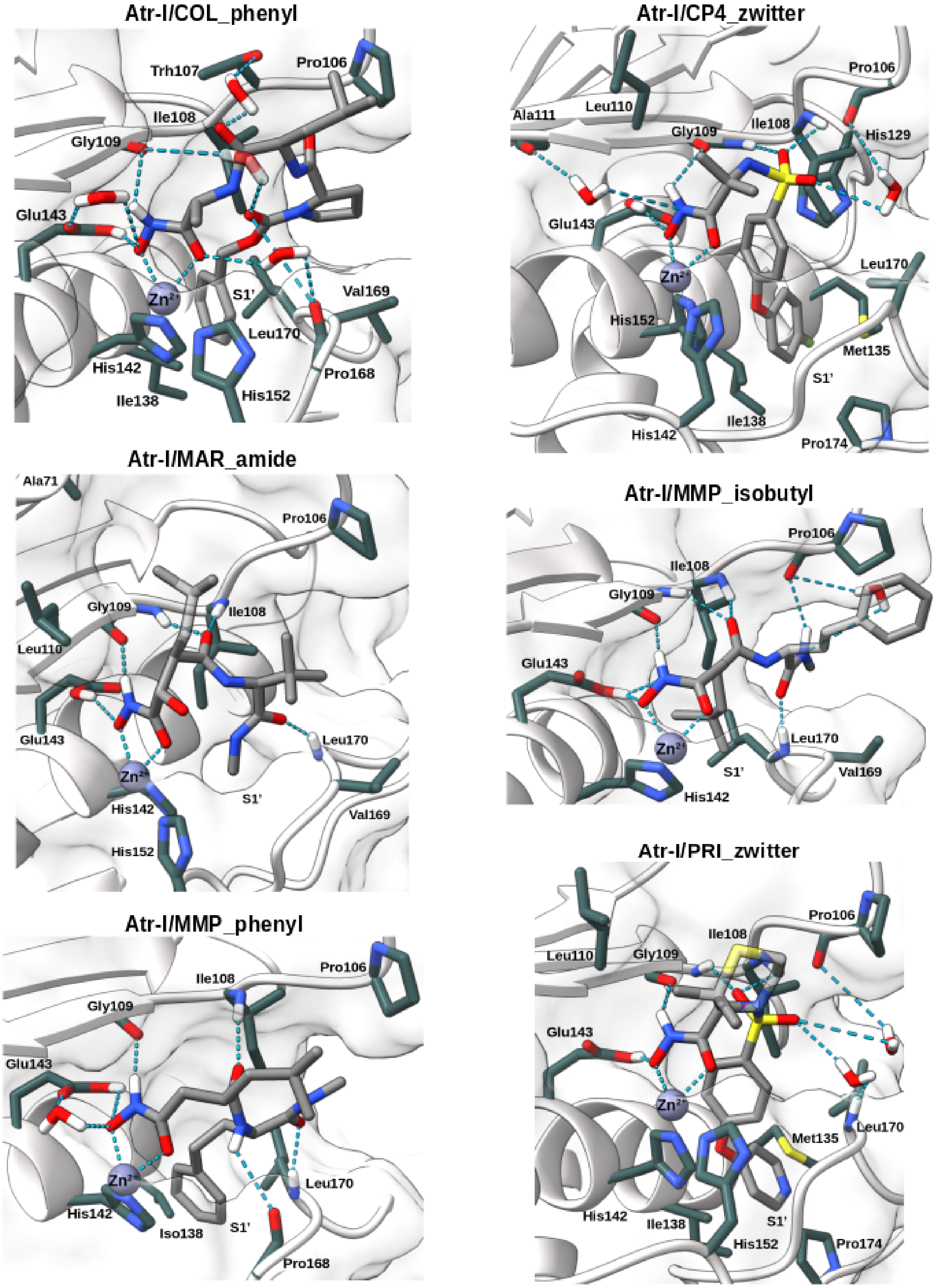

Figure S9. Part 2.

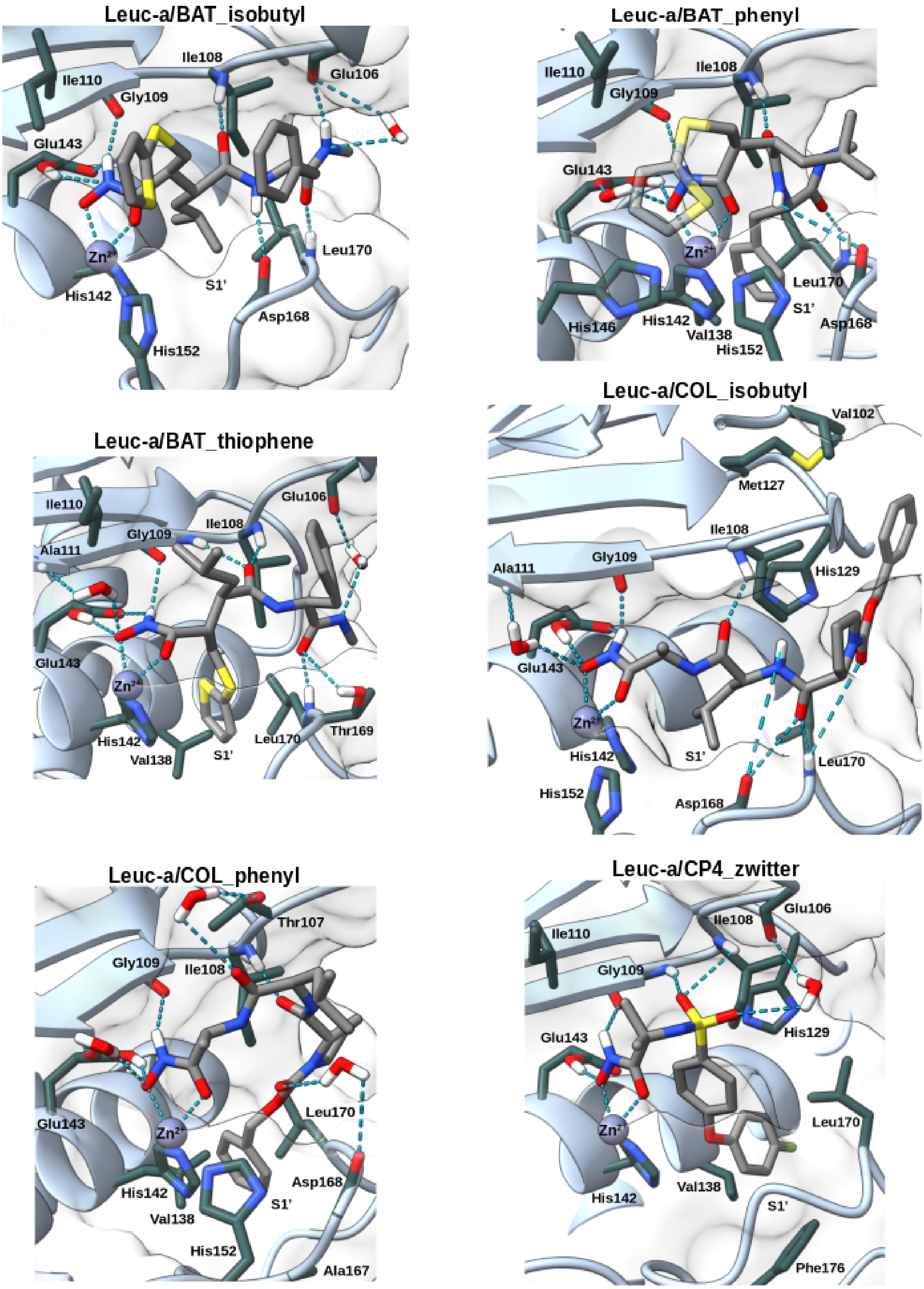

Figure S9. Part 3.

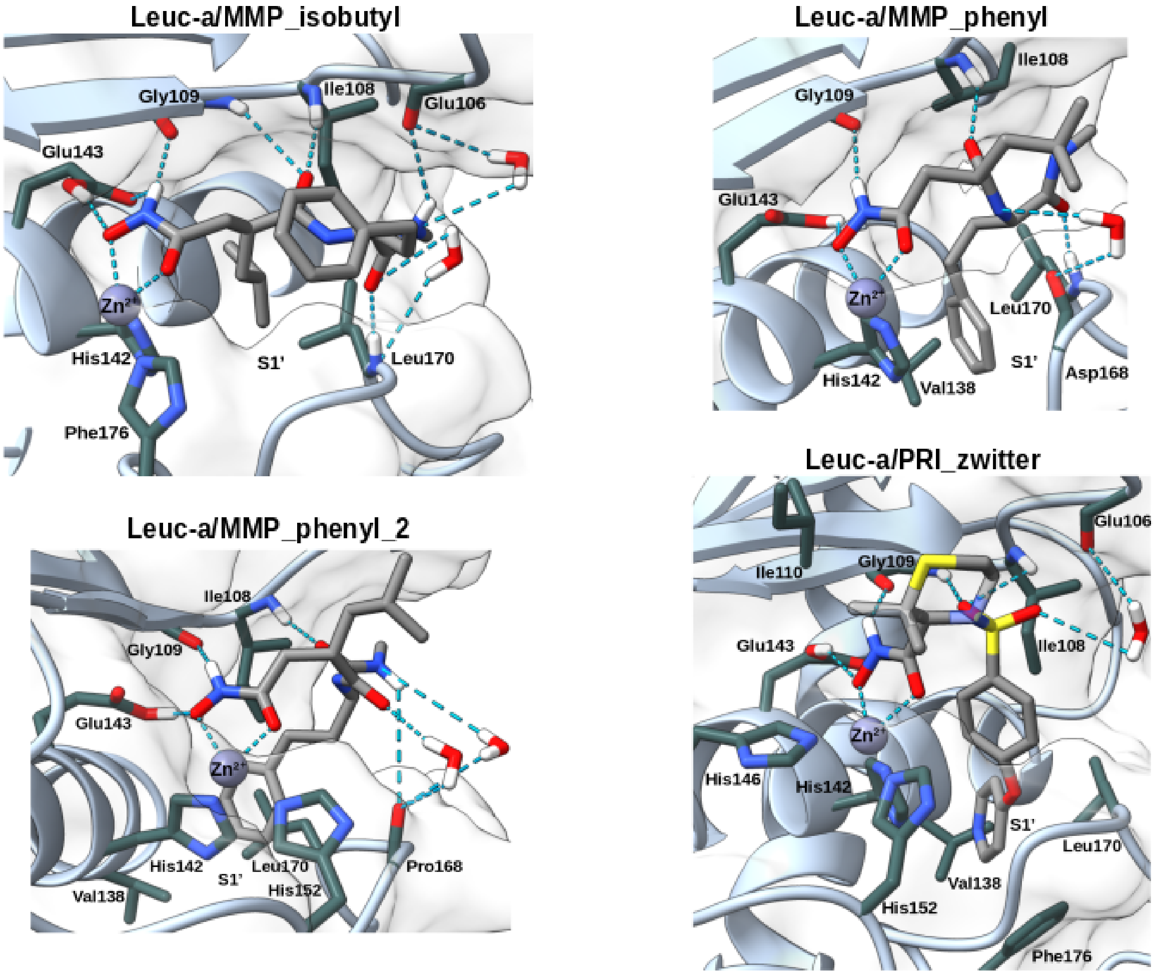

Figure S9. Part 4.

Table S4. Scoring functions evaluated and their respective *r* and *p* values of correlation tests with kinetics assays data for Atr-I and Leuc-a.

Method	Scoring Function	*r* value	*p*-value
MM/GBSA_iod	$\Delta G_{\text{int}}^{\text{trunc}} + \Delta G_{dis}^{inhi} + \Delta G_{dis}^{SVMP_{trunc}} - T(\Delta S^{(4)})$	0.78	0.003
DFTB(QM_{small})/GBSA	$\Delta G_{\text{int}} + \Delta G_{dis}^{inhi} + \Delta G_{dis}^{SVMP_{trunc}} - T(\Delta S^{(4)})$	0.78	0.003
PM6/PBSA	$\Delta G_{\text{int}}^{\text{trunc}}$	0.78	0.003
PM6/PBSA	$\Delta G_{\text{int}} + \Delta G_{dis}^{inhi}$	0.77	0.004
DFTB(QM_{small})/PBSA	$\Delta G_{\text{int}} + \Delta G_{dis}^{inhi} + \Delta G_{dis}^{SVMP_{trunc}} - T(\Delta S^{(4)})$	0.77	0.004
DFTB3/PBSA	$\Delta G_{\text{int}} - T(\Delta S^{(2)})$	0.76	0.004
PM6/PBSA	$\Delta G_{\text{int}}^{\text{trunc}} + \Delta G_{dis}^{inhi}$	0.76	0.004
DFTB(QM_{small})/PBSA	$\Delta G_{\text{int}} - T(\Delta S^{(2)})$	0.76	0.004
PM6/PBSA	$\Delta G_{\text{int}} + \Delta G_{dis}^{inhi} + \Delta G_{dis}^{SVMP_{trunc}} - T(\Delta S^{(3)})$	0.74	0.006
DFTB(QM_{small})/PBSA	$\Delta G_{\text{int}}^{\text{trunc}} + \Delta G_{dis}^{inhi} + \Delta G_{dis}^{SVMP_{trunc}} - T(\Delta S^{(4)})$	0.73	0.007
DFTB(QM_{small})/PBSA	$\Delta G_{\text{int}}^{\text{trunc}} - T(\Delta S^{(2)})$	0.73	0.007
PM6/PBSA	$\Delta G_{\text{int}}^{\text{trunc}} - T(\Delta S^{(1)})$	0.73	0.008
MMGBSA_iod	$\Delta G_{\text{int}} + \Delta G_{dis}^{inhi} + \Delta G_{dis}^{SVMP_{trunc}} - T(\Delta S^{(4)})$	0.72	0.008
DFTB(QM_{small})/GBSA	$\Delta G_{\text{int}} + \Delta G_{dis}^{inhi} + \Delta G_{dis}^{SVMP} - T(\Delta S^{(4)})$	0.72	0.008
DFTB/PBSA	$\Delta G_{\text{int}} - T(\Delta S^{(2)})$	0.72	0.009
DFTB(QM_{small})/GBSA	$\Delta G_{\text{int}}^{\text{trunc}} + \Delta G_{dis}^{inhi} - T(\Delta S^{(2)})$	0.72	0.009
PM6/PBSA	$\Delta G_{\text{int}} + \Delta G_{dis}^{inhi} - T(\Delta S^{(1)})$	0.70	0.011
DFTB(QM_{small})/GBSA	$\Delta G_{\text{int}} + \Delta G_{dis}^{inhi} - T(\Delta S^{(2)})$	0.70	0.011
DFTB(QM_{small})/PBSA	$\Delta G_{\text{int}}^{\text{trunc}} + \Delta G_{dis}^{inhi} - T(\Delta S^{(2)})$	0.70	0.011
PM6_PBSA	$\Delta G_{\text{int}}^{\text{trunc}} + \Delta G_{dis}^{inhi} + \Delta G_{dis}^{SVMP_{trunc}} - T(\Delta S^{(3)})$	0.70	0.011

The first function has the eleventh-best performance, and the *p*-value cutoff was 0.01. The “small” suffix means that only the inhibitors hydroxamate group was included in QM region.

Table S5. Statistical analysis of correlation performance variations with IC_{50} data of Atr-I showing the impact of inclusion of different energetic terms in the scoring functions.

Atr-I		*p*-values																	
		Scoring functions numbers																	
**Scoring function**	**Mean±Sd**	**1**	**2**	**3**	**4**	**5**	**6**	**7**	**8**	**9**	**10**	**11**	**12**	**13**	**14**	**15**	**16**	**17**	**18**
**1-** ΔG_{int}	0.37±0.34	-																	
**2-** $\Delta G_{int}+\Delta G^{inhi}_{dis}$	0.39±0.24	0.82	-																
**3-** $\Delta G_{int}+\Delta G^{inhi}_{dis}-T(\Delta S^{(1)})$	0.24±0.26	0.32	0.15	-															
**4-** $\Delta G_{int}+\Delta G^{inhi}_{dis}-T(\Delta S^{(2)})$	0.56±0.09	0.08	**0.04**	**1x10^{-3}**	-														
**5-** $\Delta G_{int}+\Delta G^{inhi}_{dis}+\Delta G^{SVMP}_{dis}-T(\Delta S^{(3)})$	-0.29±0.25	**3x10^{-5}**	**1x10^{-6}**	**5x10^{-5}**	**3x10^{-3}**	-													
**6-** $\Delta G_{int}+\Delta G^{inhi}_{dis}+\Delta G^{SVMP}_{dis}-T(\Delta S^{(4)})$	0.15±0.36	0.14	0.06	0.48	**2x10^{-3}**	**3x10^{-3}**	-												
**7-** $\Delta G_{int}+\Delta G^{inhi}_{dis}+\Delta G^{SVMP\_trunc}_{dis}-T(\Delta S^{(3)})$	0.33±0.31	0.78	0.57	0.45	**0.03**	**3x10^{-5}**	0.20	-											
**8-** $\Delta G_{int}+\Delta G^{inhi}_{dis}+\Delta G^{SVMP\_trunc}_{dis}-T(\Delta S^{(4)})$	0.70±0.14	**0.01**	**1x10^{-3}**	**1x10^{-5}**	**0.01**	**1x10^{-9}**	**2x10^{-4}**	**2x10^{-3}**	-										
**9 -** $\Delta G_{int}-T(\Delta S^{(1)})$	0.19±0.38	0.25	0.13	0.72	**0.01**	**2x10^{-3}**	0.77	0.34	**5x10^{-4}**	-									
**10-** $\Delta G_{int}-T(\Delta S^{(2)})$	0.63±0.19	**0.03**	**0.01**	**4x10^{-4}**	0.26	**2x10^{-9}**	**7x10^{-4}**	**0.01**	0.31	**0.002**	-								
**11-** ΔG^{trunc}_{int}	0.33±0.35	0.82	0.62	0.47	**0.05**	**6x10^{-5}**	0.21	0.98	**4x10^{-3}**	0.35	**0.02**	-							
**12 -** $\Delta G^{trunc}_{int}+\Delta G^{inhi}_{dis}$	0.38±0.23	0.87	0.94	0.16	**0.03**	**1x10^{-6}**	0.07	0.61	**6x10^{-4}**	0.14	**0.01**	0.66	-						
**13 -** $\Delta G^{trunc}_{int}+\Delta G^{inhi}_{dis}-T(\Delta S^{(1)})$	0.25±0.24	0.38	0.18	0.87	**1x10^{-3}**	**2x10^{-5}**	0.40	0.53	**3x10^{-5}**	0.62	**4x10^{-4}**	0.54	0.19	-					
**14 -** $\Delta G^{trunc}_{int}+\Delta G^{inhi}_{dis}-T(\Delta S^{(2)})$	0.53±0.12	0.14	0.10	**3x10^{-3}**	0.50	**3x10^{-8}**	**4x10^{-3}**	**0.05**	**3x10^{-3}**	**0.01**	0.14	0.08	0.07	**3x10^{-3}**	-				
**15 -** $\Delta G^{trunc}_{int}+\Delta G^{inhi}_{dis}+\Delta G^{SVMP\_trunc}_{dis}-T(\Delta S^{(3)})$	0.30±0.28	0.64	0.42	0.56	**0.01**	**2x10^{-5}**	0.25	0.85	**5x10^{-4}**	0.41	**4x10^{-4}**	0.83	0.45	0.65	**0.02**	-			
**16-** $\Delta G^{trunc}_{int}+\Delta G^{inhi}_{dis}+\Delta G^{SVMP\_trunc}_{dis}-T(\Delta S^{(4)})$	0.63±0.18	**0.03**	**0.01**	**4x10^{-4}**	0.23	**2x10^{-9}**	**7x10^{-4}**	**0.01**	0.28	**2x10^{-3}**	0.99	**0.02**	**0.01**	**3x10^{-4}**	0.12	**3x10^{-3}**	-		
**17 -** $\Delta G^{trunc}_{int}-T(\Delta S^{(1)})$	0.19±0.38	0.25	0.14	0.71	**0.01**	**2x10^{-3}**	0.79	0.34	**6x10^{-4}**	0.99	**3x10^{-3}**	0.35	0.15	0.62	**0.01**	0.41	**2x10^{-3}**	-	
**18 -** $\Delta G^{trunc}_{int}-T(\Delta S^{(2)})$	0.56±0.22	0.11	0.08	**3x10^{-3}**	0.94	**1x10^{-8}**	**3x10^{-3}**	**0.04**	0.08	**0.01**	0.44	0.06	0.06	**3x10^{-3}**	0.64	**0.02**	0.43	**0.01**	-

Mean and Standard Deviation values were computed for r values obtained in correlation analyses for each function, computed across the evaluated methods. The subsequent columns display p-values from Student's t-tests, comparing the indicated functions and highlighting p-values equal to or less than .05. Function numbers are identified in the first column.

Table S6. Statistical analysis of correlation performance variations with IC_{50} data of Leuc-a showing the impact of inclusion of different energetic terms in the scoring functions.

**Leuc-a**		***p*-values**																	
		**Scoring functions numbers**																	
**Scoring function**	**Mean±Sd**	1	2	3	4	5	6	7	8	9	10	11	12	13	14	15	16	17	18
1- ΔG_{int}	-0.15±0.44	-																	
2- $\Delta G_{\text{int}}+\Delta G_{\text{dis}}^{\text{inhi}}$	0.03±0.39	0.31	-																
3- $\Delta G_{\text{int}}+\Delta G_{\text{dis}}^{\text{inhi}}-T(\Delta S^{(1)})$	0.05±0.34	0.23	0.89	-															
4- $\Delta G_{\text{int}}+\Delta G_{\text{dis}}^{\text{inhi}}-T(\Delta S^{(2)})$	0.60±0.25	**0.00**	**0.00**	**0.00**	-														
5- $\Delta G_{\text{int}}+\Delta G_{\text{dis}}^{\text{inhi}}+\Delta G_{\text{dis}}^{\text{SVMP}}-T(\Delta S^{(3)})$	0.10±0.41	0.16	0.65	0.74	**0.00**	-													
6- $\Delta G_{\text{int}}+\Delta G_{\text{dis}}^{\text{inhi}}+\Delta G_{\text{dis}}^{\text{SVMP}}-T(\Delta S^{(4)})$	0.54±0.33	**0.00**	**0.00**	**0.00**	0.63	**0.01**	-												
7- $\Delta G_{\text{int}}+\Delta G_{\text{dis}}^{\text{inhi}}+\Delta G_{\text{dis}}^{\text{SVMP\_trunc}}-T(\Delta S^{(3)})$	0.04±0.33	0.26	0.96	0.92	**0.00**	0.67	**0.00**	-											
8- $\Delta G_{\text{int}}+\Delta G_{\text{dis}}^{\text{inhi}}+\Delta G_{\text{dis}}^{\text{SVMP\_trunc}}-T(\Delta S^{(4)})$	0.47±0.29	**0.00**	**0.00**	**0.00**	0.26	**0.02**	0.59	**0.00**	-										
9 - $\Delta G_{\text{int}}-T(\Delta S^{(1)})$	-0.05±0.36	0.58	0.59	0.47	**0.00**	0.32	**0.00**	0.52	**0.00**	-									
10-$\Delta G_{\text{int}}-T(\Delta S^{(2)})$	0.47±0.31	**0.00**	**0.01**	**0.00**	0.26	**0.02**	0.58	**0.00**	0.97	**0.00**	-								
11- $\Delta G_{\text{int}}^{\text{trunc}}$	-0.07±0.52	0.70	0.60	0.51	**0.00**	0.37	**0.00**	0.55	**0.01**	0.94	**0.01**	-							
12 - $\Delta G_{\text{int}}^{\text{trunc}}+\Delta G_{\text{dis}}^{\text{inhi}}$	0.08±0.48	0.24	0.77	0.85	**0.00**	0.91	**0.01**	0.79	**0.03**	0.44	**0.03**	0.46	-						
13 - $\Delta G_{\text{int}}^{\text{trunc}}+\Delta G_{\text{dis}}^{\text{inhi}}-T(\Delta S^{(1)})$	0.10±0.42	0.17	0.65	0.73	**0.00**	0.99	**0.01**	0.67	**0.02**	0.33	**0.03**	0.37	0.90	-					
14 - $\Delta G_{\text{int}}^{\text{trunc}}+\Delta G_{\text{dis}}^{\text{inhi}}-T(\Delta S^{(2)})$	0.55±0.31	**0.00**	**0.00**	**0.00**	0.68	**0.01**	0.93	**0.00**	0.52	**0.00**	0.51	**0.00**	**0.01**	**0.01**	-				
15 - $\Delta G_{\text{int}}^{\text{trunc}}+\Delta G_{\text{dis}}^{\text{inhi}}+\Delta G_{\text{dis}}^{\text{SVMP\_trunc}}-T(\Delta S^{(3)})$	0.11±0.39	0.14	0.60	0.68	**0.00**	0.95	**0.01**	0.61	**0.02**	0.28	**0.02**	0.34	0.86	0.96	**0.01**	-			
16- $\Delta G_{\text{int}}^{\text{trunc}}+\Delta G_{\text{dis}}^{\text{inhi}}+\Delta G_{\text{dis}}^{\text{SVMP\_trunc}}-T(\Delta S^{(4)})$	0.50±0.36	**0.00**	**0.00**	**0.00**	0.46	**0.02**	0.80	**0.00**	0.81	**0.00**	0.79	**0.00**	**0.02**	**0.02**	0.73	**0.02**	-		
17 - $\Delta G_{\text{int}}^{\text{trunc}}-T(\Delta S^{(1)})$	0.03±0.44	0.34	0.99	0.88	**0.00**	0.66	**0.00**	0.95	**0.01**	0.62	**0.01**	0.63	0.77	0.66	**0.00**	0.61	**0.01**	-	
18 - $\Delta G_{\text{int}}^{\text{trunc}}-T(\Delta S^{(2)})$	0.56±0.32	**0.00**	**0.00**	**0.00**	0.71	**0.01**	0.92	**0.00**	0.51	**0.00**	0.50	**0.00**	**0.01**	**0.01**	0.98	**0.01**	0.72	**0.00**	-

Mean and Standard Deviation values were computed for r values obtained in correlation analyses for each function, computed across the evaluated methods. The subsequent columns display p-values from Student's t-tests, comparing the indicated functions and highlighting p-values equal to or less than .05. Function numbers are identified in the first column.

Table S7. Statistical analysis of correlation performance variations with IC_{50} data of Atr-I showing the impact of the use of different energetic methods in the scoring functions.

Atr-I		*p*-values											
		Energy methods numbers											
**Energy Methods**	**Mean±Sd**	**1**	**2**	**3**	**4**	**5**	**6**	**7**	**8**	**9**	**10**	**11**	**12**
1 - DFTB3/GBSA	0.38±0.19	-											
2 - DFTB3/PBSA	0.32±0.33	0.52	-										
3 - MM/GBSA_hfe	0.40±0.33	0.82	0.48	-									
4 - MM/GBSA_iod	0.39±0.33	0.85	0.50	0.98	-								
5 - MM/PBSA_hfe	0.03±0.32	**$6x10^{-4}$**	**0.01**	**$2x10^{-3}$**	**$2x10^{-3}$**	-							
6 - MM/PBSA_iod	0.03±0.33	**$7x10^{-4}$**	**0.01**	**$2x10^{-3}$**	**$2x10^{-3}$**	0.99	-						
7 - PM6/GBSA	0.33±0.29	0.60	0.89	0.54	0.56	**0.01**	**0.01**	-					
8 - PM6/PBSA	0.63±0.33	**0.01**	**0.01**	**0.04**	**0.04**	**$4x10^{-6}$**	**$4x10^{-6}$**	**0.01**	-				
9 - DFTB/GBSA	0.27±0.25	0.15	0.61	0.19	0.21	**0.02**	**0.02**	0.48	**$7x10^{-4}$**	-			
10 - DFTB/GBSA_small	0.59±0.16	**$7x10^{-4}$**	**$4x10^{-3}$**	**0.03**	**0.03**	**$8x10^{-7}$**	**$1x10^{-6}$**	**$3x10^{-3}$**	0.59	**$7x10^{-5}$**	-		
11 - DFTB/PBSA	0.39±0.39	0.88	0.54	0.97	0.99	**0.01**	**0.01**	0.61	**0.05**	0.26	0.06	-	
12 - DFTB/PBSA_small	0.55±0.26	**0.03**	**0.03**	0.14	0.13	**0.01**	**$1x10^{-5}$**	**0.03**	0.38	0.27	0.56	0.17	-

Mean and Standard Deviation values were computed for *r* values obtained in correlation analyses for each energy method, computed across the evaluated scoring functions. The subsequent columns display *p*-values from Student's *t*-tests, comparing the indicated methods and highlighting values equal to or less than .05. Methods numbers are identified in the first column.

Table S8. Statistical analysis of correlation performance variations with IC_{50} data of Leuc-a showing the impact of the use of different energetic methods in the scoring functions.

**Leuc-a**		***p*-values**											
		**Energy Methods numbers**											
**Energy Methods**	**Mean±Sd**	**1**	**2**	**3**	**4**	**5**	**6**	**7**	**8**	**9**	**10**	**11**	**12**
1 - DFTB3/GBSA	0.46±0.33	-											
2 - DFTB3/PBSA	0.45±0.25	0.92	-										
3 - MM/GBSA_hfe	-0.03±0.43	**0.00**	**0.00**	-									
4 - MM/GBSA_iod	0.10±0.44	**0.01**	**0.01**	0.37	-								
5 - MM/PBSA_hfe	-0.26±0.29	**0.00**	**0.00**	0.07	**0.01**	-							
6 - MM/PBSA_iod	-0.14±0.41	**0.00**	**0.00**	0.45	0.10	0.29	-						
7 - PM6/GBSA	0.15±0.35	**0.01**	**0.01**	0.17	0.72	**0.00**	**0.03**	-					
8 - PM6/PBSA	0.73±0.09	**0.00**	**0.00**	**0.00**	**0.00**	**0.00**	**0.00**	**0.00**	-				
9 - DFTB/GBSA	-0.01±0.32	**0.00**	**0.00**	0.87	0.38	**0.02**	0.30	0.16	**0.00**	-			
10 - DFTB/GBSA_small	0.59±0.20	0.17	0.07	**0.00**	**0.00**	**0.00**	**0.00**	**0.00**	**0.01**	**0.00**	-		
11 - DFTB/PBSA	-0.02±0.30	**0.00**	**0.00**	0.94	0.33	**0.02**	0.34	0.12	**0.00**	0.91	**0.00**	-	
12 - DFTB/PBSA_small	0.61±0.19	0.11	**0.04**	**0.00**	**0.00**	**0.00**	**0.00**	**0.00**	**0.03**	**0.00**	0.75	**0.00**	-

Mean and Standard Deviation values were computed for r values obtained in correlation analyses for each energy method, computed across the evaluated scoring functions. The subsequent columns display p-values from Student's t-tests, comparing the indicated methods and highlighting values equal to or less than .05. Methods numbers are identified in the first column.
